# Women with moderate anaemia prior to conception benefited most from nutrition interventions: a secondary analysis of the Women First preconception maternal nutrition trial

**DOI:** 10.1136/bmjgh-2025-020160

**Published:** 2026-01-23

**Authors:** Sumera Aziz Ali, Ka Kahe, Jeanine M Genkinger, Linda Valeri, Sarah Saleem, Saleem Jessani, Robert L Goldenberg, Jamie Westcott, Jennifer Kemp, Ana Garces, Lester Figueroa, Shivaprasad S Goudar, Sangappa M Dhaded, Richard Derman, Antoinette Tshefu Kitoto, Adrien Lokangaka, Melissa Bauserman, Elizabeth M McClure, Marion Koso-Thomas, Louise Kuhn, Nancy F Krebs, Women First Preconception Nutrition Trial Group, Omrana Pasha Razzak

**Affiliations:** 1 Department of Pediatrics, University of Alberta, Edmonton, Alberta, Canada; 2 Department of Epidemiology, Columbia University Mailman School of Public Health, New York, New York, USA; 3 Department of Obstetrics and Gynecology, New York-Presbyterian/Columbia University Irving Medical Center, New York, New York, USA; 4 Columbia University Herbert Irving Comprehensive Cancer Center, New York, New York, USA; 5 Department of Biostatistics, Columbia University Mailman School of Public Health, New York, New York, USA; 6 Department of Epidemiology, Harvard T.H. Chan School of Public Health, Boston, Massachusetts, USA; 7 Department of Community Health Sciences, The Aga Khan University, Karachi, Sindh, Pakistan; 8 Department of Pediatrics, Section of Nutrition, University of Colorado Denver Anschutz Medical Campus, Aurora, Colorado, USA; 9 Institute of Nutrition of Central America and Panama, Guatemala City, Guatemala; 10 Women's and Children’s Health Research Unit, KLE Academy of Higher Education & Research Deemed to be University - JNMC Campus, Belagavi, KA, India; 11 Thomas Jefferson University, Philadelphia, Pennsylvania, USA; 12 University of Kinshasa, Kinshasa, Congo (the Democratic Republic of the); 13 Department of Pediatrics, The University of North Carolina at Chapel Hill, Chapel Hill, North Carolina, USA; 14 Research Triangle Institute, Research Triangle Park, North Carolina, USA; 15 National Institute of Child Health and Human Development, Bethesda, Maryland, USA; 16 Vagelos College of Physicians and Surgeons, Columbia University Gertrude H Sergievsky Center, New York, New York, USA

**Keywords:** Global Health, Maternal health, Nutrition, Anaemia, Pregnancy

## Abstract

**Introduction:**

The Women First (WF) Preconception Maternal Nutrition trial found greater benefits of small-quantity lipid-based nutrient supplements (SQ-LNS) for intrauterine growth among anaemic versus non-anaemic women at preconception. We investigated whether the benefits of SQ-LNS in improving markers of intrauterine growth occurred evenly across the mild to moderate spectrum of pre-pregnancy anaemia.

**Methods:**

We analysed WF data (n=2443 maternal-newborn dyads) from Pakistan, India, Guatemala and the Democratic Republic of Congo. Women received SQ-LNS either ≥3 months preconception through pregnancy (Arm 1); starting in the late first trimester (Arm 2); or not at all (Arm 3: control), with all supplementations discontinued at delivery. The outcomes were infant weight, length and head circumference measured within 48 hours of birth, expressed as Z-scores. For each site, adjusted mean differences in the Z-scores were computed across six pre-pregnancy haemoglobin (Hb) categories (80–89, 90–99, 100–109, 110–119, 120–129, and ≥130 g/L) and pooled using meta-analysis.

**Results:**

The effect of SQ-LNS on birth weight, length and head circumference varied by pre-pregnancy Hb categories. No significant differences in pooled mean Z-scores were observed for any Hb category >110 g/L, and no differences were found for Arm 1 vs Arm 2 across any Hb categories. For women with Hb 90–99 g/L pooled mean differences (95% CI) in the Z-scores for length (0.60 (0.03 to 1.23)), weight (0.50 (0.11 to 0.89)) and head circumference (0.26 (0.02 to 0.51)) were greatest for Arm 1 versus Arm 3. For women with Hb 100–109 g/L in Arm 1 versus Arm 3, pooled mean difference (95% CI) in birth weight Z-scores was significantly greater (0.33 (0.24 to 0.42)). Arm 2 vs Arm 3 women with Hb 90–99 g/L had greater birth weight Z-scores (0.14 (0.05 to 0.22)).

**Conclusion:**

The findings highlight the importance of identifying women preconception for whom nutrition interventions may have the greatest impact on fetal growth.

**WHAT IS ALREADY KNOWN ON THIS TOPIC**

- The Women First (WF) Preconception Maternal Nutrition Trial documented that small-quantity lipid-based nutrient supplementation (SQ-LNS) initiated either ≥3 months preconception or during early pregnancy improved birth weight and length outcomes more effectively in anaemic women (haemoglobin (Hb) Hb <120 g/L) compared with non-anaemic women (Hb ≥120 g/L) prior to pregnancy.

**WHAT THIS STUDY ADDS**

- This study reveals that the positive effects of preconception SQ-LNS supplementation on fetal growth indicators were most evident in women with moderate anaemia before pregnancy, with no significant benefits observed in women with mild anaemia.
- These results highlight the importance of prioritising women with at least moderate anaemia for preconception nutrition interventions.

**HOW THIS STUDY MIGHT AFFECT RESEARCH, PRACTICE OR POLICY**

- Using point-of-care devices to assess pre-pregnancy Hb levels could provide a practical and cost-effective method for identifying high-risk women in low- and middle-income countries (LMICs), enabling more efficient allocation of nutrition resources.
- There is a need for further research with larger sample sizes to assess these effects more comprehensively, particularly for women with lower Hb concentrations.

## Introduction

 The Women First (WF) Preconception Maternal Nutrition Trial found that multiple micronutrient-fortified small-quantity lipid-based nutrient supplementation (SQ-LNS), initiated at least 3 months before conception or during early pregnancy, provided greater benefits for both birth weight and length among mothers who were anaemic (haemoglobin (Hb) < 120 g/L) compared with those who were non-anaemic (Hb≥120 g/L) prior to pregnancy.^1^ This finding is consistent with a meta-analysis on effect modifiers of nutrition supplements and birth outcomes, which reported that providing micronutrient supplements to anaemic women resulted in a greater reduction in the risk of small-for-gestational-age and low birth weight than providing the same supplements to non-anaemic women.^2^

This prior research investigating maternal anaemia as an effect modifier dichotomised women into anaemic (Hb<120 g/L) and non-anaemic (Hb≥120 g/L) categories,^1 2^ limiting our understanding of whether the effects of nutrient supplementation on intrauterine growth vary across the spectrum of pre-pregnancy Hb concentrations, ranging from moderate to mild anaemia. Among women with Hb <120 g/L, those with Hb between 110 and 119 g/L are classified as having mild anaemia, while those with Hb between 80 and 109 g/L are considered to have moderate anaemia and those with Hb <80 g/L are classified as having severe anaemia.^3^ Women with Hb <80 g/L were excluded from the WF Preconception Maternal Nutrition trial. By categorising women simply into anaemic and non-anaemic groups, we may miss high-risk women with specific Hb values who may benefit most from nutrient supplements. Assessing the effect of the SQ-LNS across the mild to moderate spectrum of pre-pregnancy anaemia may help identify women who have the greatest potential to respond to nutrition interventions.

In this secondary analysis, we used data from the WF Preconception Maternal Nutrition trial^4 5^ to investigate whether the effects of initiating multiple micronutrient-fortified SQ-LNS at least 3 months prior to conception or late in the first trimester on intrauterine growth vary across the mild to moderate spectrum of pre-pregnancy anaemia. We investigated whether benefits accrued across three birth anthropometric indicators (weight, length and head circumference) and whether the patterns of association persisted after adjusting for gestational age (GA).

## Methods

### Study design, setting and study participants

We performed a secondary analysis of an individual randomised controlled trial, the WF Preconception Maternal Nutrition Trial, with three arms.^4 5^ All women were enrolled and randomised prior to conception. Participants in Arm 1 consumed the SQ-LNS daily for ≥3 months before pregnancy and continued the SQ-LNS until delivery. Women in Arm 2 consumed the SQ-LNS from the end of the first trimester of pregnancy until delivery. Women in Arm 3 (control) did not receive SQ-LNS from the study but received iron-folate supplements according to the country’s standard of care.^4 5^ The trial was undertaken concurrently in rural areas of Pakistan, India, the Democratic Republic of the Congo (DRC) and Guatemala.^4 5^ The randomisation of participants to three arms was done in 53 clusters or geographic catchment areas in the four countries.^6^ Participants enrolled in the trial were 16–35 years old with Hb of ≥80 g/L and were planning to conceive during the following 18 months.^4 5^ All women provided written informed consent.^4 5^ The Colorado Multiple Institutional Review Board and internal review boards of the participating institutions in the four countries and the data coordinating centre (DCC) (RTI International) approved the trial. The trial is registered with clinicaltrials.gov (NCT01883193).^4 5^

### Randomisation and masking

The DCC generated the randomisation scheme and created the allocation sequence for each country. The DCC used a permuted block design with a stratification by geographic clusters to ensure balance of women across three arms and 53 geographic clusters.^4 5^ Women were randomised to the three arms with 1:1:1 allocation ratio within randomly varied blocks of 3, 6 or 9 sizes for each country. With the help of a centralised data management system maintained by the DCC, a country-specific data manager generated the random assignment for each woman identified by the data collector in geographic cluster.^4 5^ While women and research investigators were not masked to the assigned intervention, the assessment team responsible for measuring the outcome of interest was blinded to the intervention assignment.^4 5^

### Primary intervention and compliance

The intervention for the WF trial consisted of a daily 20 g sachet of SQ-LNS providing 20 mg of iron and 400 µg of folic acid, along with multiple vitamins and minerals (including iodine, copper, calcium, phosphorus, magnesium, potassium, selenium and zinc), proteins, polyunsaturated fatty acids and mono-glycerides (118 kcal per sachet).^4 5^ Participants in Arm 3 (control) received usual care at each site, in line with local practice for antenatal supplementation, which typically consisted of daily iron–folic acid (IFA) supplementation, providing approximately 60 to 100 mg of elemental iron and 400 µg of folic acid per day. Compliance with the SQ-LNS was estimated as the number of sachets fully consumed by women divided by the number of days between commencing the SQ-LNS and the birth of a baby. Compliance with the SQ-LNS was 87.2% and 84.3% for Arm 1 and Arm 2, respectively, as previously published by the Women First research group.^4^ While overall compliance was high, we additionally examined adherence by pre-pregnancy Hb categories. In Arm 1, mean compliance ranged from 83.8% among women with Hb ≥130 g/L to 91.6% among those with Hb 90–99 g/L, with slightly lower compliance (87.4%) in the smallest Hb 80–89 g/L subgroup. In Arm 2, mean compliance ranged from 76.0% in women with Hb ≥130 g/L to 88.8% in those with Hb 100–109 g/L and 86.0% in the Hb 80–89 g/L subgroup. These results indicate that adherence was generally high (≥80%) across all anaemia categories, with no consistent patterns suggesting differential compliance by baseline Hb.

### Outcome of interest

Since the WF trial focused on the impact of nutritional interventions on intrauterine growth, we selected three key anthropometric markers—birth weight, length and head circumference—that are well-established indicators of fetal growth and intrauterine development.^7 8^ In the WF study, birth weight, length and head circumference of the newborns were measured within 48 hours of birth. For the current analysis, the anthropometric outcome measures used were Z-scores calculated using the WHO child growth standards that account for the newborn’s sex and chronological age at measurement.^9^ To investigate directly whether these markers were describing intrauterine growth and not confounded by GA, we performed additional analysis for Pakistan, India and Guatemala using sex and GA-adjusted Z-scores based on the INTERGROWTH-21st international fetal growth standards.^10 11^ GA was determined using first-trimester ultrasound dating whenever available, primarily by measuring fetal crown-rump length between 6 and 13 weeks of gestation.^4 5^ The limitation in GA data availability arose from challenges in obtaining early pregnancy ultrasound data, particularly in the DRC, where GA could not be determined due to a lack of ultrasound capacity and trained personnel.^4^ This resulted in the exclusion of the sub-Saharan African site from the GA analysis. Additionally, the proportion of available GA data was reduced to 69%–86% due to the incomplete availability of first-trimester ultrasound data for all participants across the other three sites, particularly in Pakistan.^4^

### Proposed effect modifier

Hb before pregnancy was a hypothesised effect modifier for the association between preconception and during-pregnancy SQ-LNS and three markers of intrauterine growth. Women’s Hb concentrations (g/L) were measured using Sahli’s method in India^12^ and calibrated HemoCue devices (HemoCue Hb 201^+^ System; HemoCue America, Brea, CA 92821) in Pakistan, DRC and Guatemala.^13^,^15^ Following standard protocols, capillary blood samples were obtained from all study participants at the time of screening prior to the enrolment and initiation of study procedures.^4 5^ Women in Arm 1 (preconception-to-birth SQ-LNS) were provided the nutritional intervention during the preconception period, with pregnancy occurring between 3 and 18 months after enrolment.^4 5^ Site-specific training was conducted to reinforce proper fingerstick techniques, ensuring that excess interstitial fluid was not introduced, which could affect the accuracy and reliability of the Hb measurements. Additionally, lancets and blood collection procedures were standardised across all sites, reducing variability and ensuring uniformity in data collection. These measures were critical in maintaining the reliability of Hb measurements throughout the study. Adjustments for external factors influencing Hb concentrations, such as elevation and smoking, were considered. Since less than 1% of women reported smoking in the WF study, no adjustments were made. However, Hb results were adjusted according to WHO guidelines for altitude for the Guatemala site, where elevations were between 1000 and 2000 m. Women with Hb of <80 g/L were excluded because they needed special treatment; hence, severe anaemia (< 80 g/L) could not be explored. We grouped women into six Hb categories (80–89, 90–99, 100–109, 110–119, 120–129, and ≥130 g/L) to assess the effect measure modification across pre-pregnancy Hb.

### Other potential variables

Baseline data collected at enrolment were available for sociodemographic variables including maternal age (years), parity, education and body mass index (BMI, kg/m^2^). Using WHO cut-offs for non-pregnant women for reproductive age, we categorised women as underweight (BMI <18.5 kg/m^2^), healthy weight (18.5–24.9 kg/m^2^) and overweight or obese (≥ 25.0 kg/m^2^). The data on economic indicators such as electricity, improved water source, man-made flooring, improved cooking fuels, sanitation and household assets were used to generate the variable for socioeconomic status (SES).^1 4^

### Study participants and analytical sample

Online supplemental figure 1 illustrates the flow of study participants in the WF trial by intervention arm. As previously published,^4^ 12 551 women were screened in the WF trial, 61.2% (n=7686) were eligible, and 96.1% of the eligible women (n=7387) were randomised after obtaining their written informed consent.^4^ Of the randomised women, 44% (n=3251) conceived within 3–18 months of enrolment. Data on the birth outcomes were recorded for 97.2% of pregnant women (n=3163), including 25 multiple births (n=3188 newborns).^4^ There were 520 miscarriages, 82 stillbirths and 2586 live births. Weight, length and head circumference were measured within 48 hours of birth for 94.5% (n=2443). Pre-pregnancy Hb data were available for 100% of women with live births. Thus, our analysis included 2443 maternal-newborn dyads (India: 590; Pakistan: 663; Guatemala: 613; and DRC: 577) with complete data on Hb and birth weight, length and head circumference. Ultrasound-determined GA data were available for 85.9% of maternal-newborn dyads from India (n=507), 68.6% from Pakistan (n=456) and 80.4% from Guatemala (n=493), thereby reducing the sample size to 1456 for the GA adjusted analyses of intrauterine growth.

### Statistical analysis

After assessing the distribution of continuous variables using histograms and p-p plots, we computed means with SD for maternal age, BMI and Hb for each site. We generated site-specific frequencies (n) and corresponding proportions (%) for education, parity and SES. We used the χ^2^ test to report frequencies and proportions of the baseline categorical variables across the three arms individually for each site. The analysis of variance (ANOVA) test was used to compare means across the three arms for continuous variables. Since diet and culture in the four countries are substantially different,^16^ we analysed data within each country, followed by pooling country-specific results with meta-analysis for effect measure modification by pre-pregnancy Hb. We quantified heterogeneity across four countries using *I*^2^ statistics.^17^ Prior to pooling results, we performed country-specific analyses by employing generalised estimating equations exchangeable correlation matrix^18^ accounting for within-cluster correlations and adjusting for maternal age, parity, education, BMI and SES. Since there were three intervention arms, three anthropometric markers and six Hb categories, we adjusted for multiple testing using Bonferroni correction method^19^ and compared the site-specific effect measure modification findings against the alpha threshold of 0.0009. To estimate 95% CIs for adjusted mean differences in Z-scores between the randomised arms, we used robust standard errors. We performed analyses using SAS version 9.4 (SAS Institute, Cary, NC, USA) and R version 4.3.1.

### Patient and Public Involvement

No patients or members of the public were involved in the design, conduct, reporting or dissemination of this research.

## Results

### Distribution of pre-pregnancy haemoglobin across sites and arms

For the combined sites, the sample sizes according to Hb category (g/L) were 168 (80–89), 305 (90–99), 444 (100–109), 453 (110–119), 427 (120–129) and 646 (≥ 130). The pooled mean±SD pre-pregnancy Hb was similar across all three arms (Arm 1: Hb 117±13.8 g/L; Arm 2: 118±13.2 g/L; and Arm 3: 117 ± 12.7 g/L).

**Figure 1:**
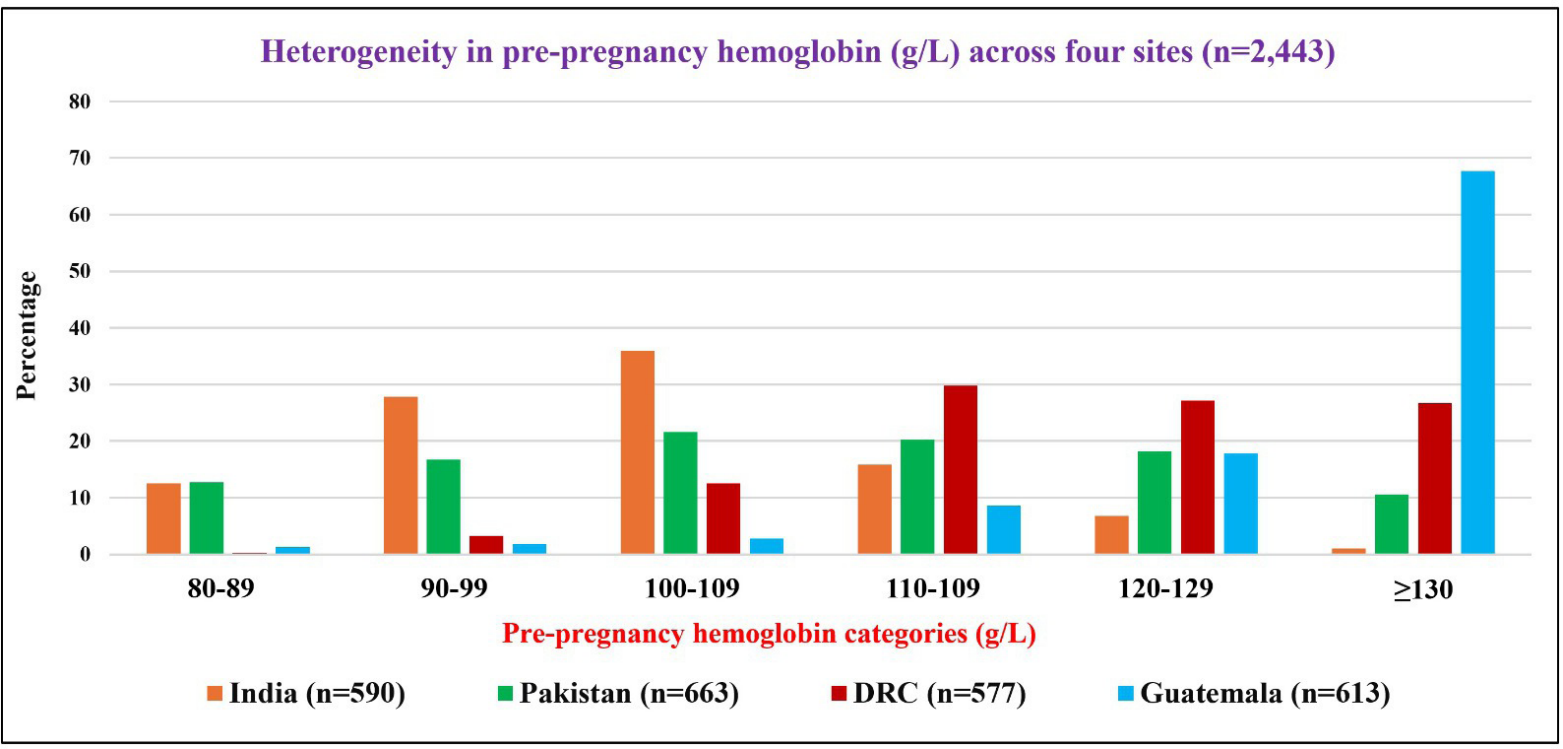
Distribution of pre-pregnancy haemoglobin (g/L) across four participating sites (India, Pakistan, Democratic Republic of Congo (DRC) and Guatemala) in the Women First Preconception Maternal Nutrition Trial.

There was substantial heterogeneity in pre-pregnancy Hb levels across sites (figure 1). Anaemia was most prevalent in India, where most women had Hb <120 g/L, followed by Pakistan, DRC and Guatemala. Moderate anaemia (Hb: 80–109 g/L) was particularly more frequent in India and Pakistan than in DRC and Guatemala. Notably, a large proportion of Indian women had Hb values clustered around 90–109 g/L, while in Pakistan, Hb levels tended to be slightly higher, with many women falling within the 100–119 g/L range. For each site, there were no detectable differences in baseline characteristics by arm (online supplemental Tables 1 and 2). The distribution of potential baseline maternal characteristics by pre-pregnancy Hb categories is shown in online supplemental Table 3.

### Effect of SQ-LNS on birth anthropometric indicators stratified by pre-pregnancy Hb (g/dL) concentrations

#### Non-GA-adjusted anthropometric data

**Figure 2:**
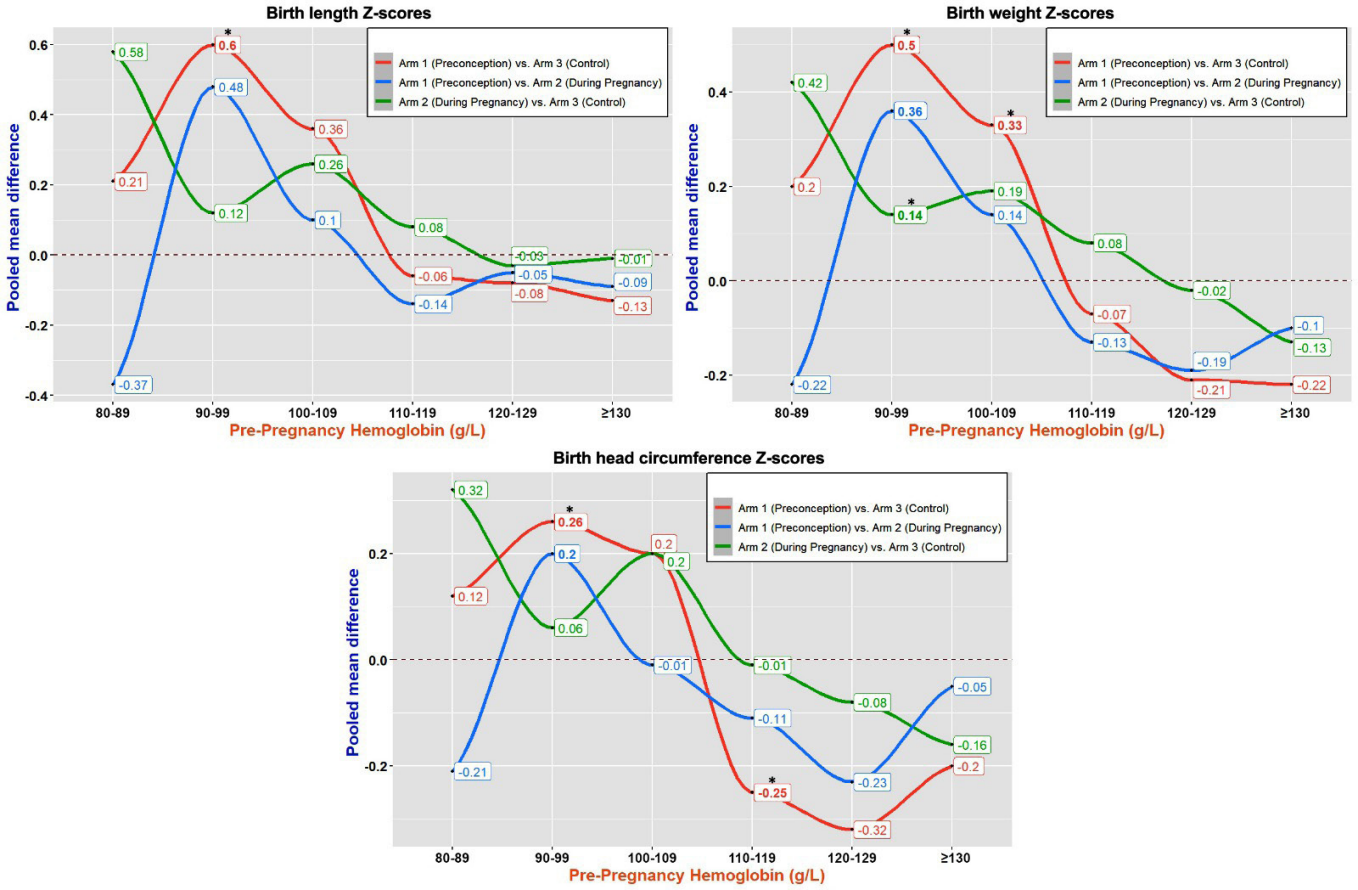
Effect of small-quantity lipid-based nutrient supplement on three birth anthropometric indicators stratified by pre-pregnancy haemoglobin (g/L) showing Arm wise comparison in Z-scores for birth length, weight and head circumference after pooling site-specific mean differences in the three Z-scores from Pakistan, India, Guatemala and Democratic Republic of Congo (DRC) using data from online supplemental Table 4 (WHO child growth standards: non-gestational age-adjusted data).Arm 1: Received nutrition supplement from preconception to delivery; Arm 2: Nutrition supplement only during pregnancy (end of the first trimester until delivery); Arm 3: No nutrition supplement (control arm). The bold pooled mean differences (beta coefficients) in Z-scores with asterisks (*) are statistically significant. See Table 1 for sample size in each Hb (g/dL) category and 95% CIs for pooled mean difference in Z-scores across three arms

The effect of SQ-LNS on birth anthropometric indicators varied by pre-pregnancy Hb concentrations. Pooled mean differences (95% CI) in the Z-scores for birth length (0.60 (0.03 to 1.23)), weight (0.50 (0.11 to 0.89)) and head circumference (0.26 (0.02 to 0.51)) were greatest for Arm 1 (preconception-to-birth SQ-LNS) versus Arm 3 (control) women with Hb 90–99 g/L (figure 2 and table 1). Compared with those in Arm 3, for women in Arm 1 with Hb 100–109 g/L, the pooled mean differences (95% CI) in the Z-scores were significantly greater for birth weight (0.33 (0.24 to 0.42)) only. Pooled mean differences in the three Z-scores for Arm one vs Arm 3 women with Hb ≥110 g/L were generally negative and not statistically significant (figure 2 and table 1). Site-specific results revealed a similar pattern, although with varying effect sizes (online supplemental Table 4).

**Table 1:** Pooled mean difference (95% CIs) in Z-scores for birth length, weight and head circumference-for-age between randomised arms: Stratified by pre-pregnancy haemoglobin (g/L) categories- (*non-gestational age adjusted data: WHO child growth standards, n=2443*)

Markers of intrauterine growth	Arm 1	Arm 2	Arm 3	Arm one vs Arm 3	Arm one vs Arm 2	Arm two vs Arm 3
	Pooled means±SD			Pooled mean differences in Z-scores* (95% CIs)		
Birth length-for-age Z-scores						
Hb: 80–89 g/L	−1.19±1.11	−0.83±1.02	−1.39±1.25	0.21 (−2.07 to 2.49)	−0.37 (−3.24 to 2.53)	0.58 (−0.29 to 1.44)
Hb: 90–99 g/L	−0.84±1.22	−1.32±1.45	−1.43±1.45	**0.60 (0.03 to 1.23**)	0.48 (−0.38 to 1.16)	0.12 (−0.16 to 0.40)
Hb: 100–109 g/L	−0.92±1.20	−1.01±1.12	−1.28±1.17	0.36 (−0.26 to 0.73)	0.10 (−0.78 to 0.97)	0.26 (−0.17 to 0.70)
Hb: 110–119 g/L	−1.13±1.15	−1.00±1.09	−1.09±1.16	−0.06 (−0.41 to 0.28)	−0.14 (−0.32 to 0.22)	0.08 (−0.27 to 0.45)
Hb: 120–129 g/L	−1.16±1.33	−1.11±1.06	−1.08±1.10	−0.08 (−0.50 to 0.34)	−0.05 (−0.53 to 0.40)	−0.03 (−0.20 to 0.10)
Hb≥130 g/L	−1.23±1.18	−1.15±1.01	−1.10±1.02	−0.13 (−0.54 to 0.28)	−0.09 (−0.44 to 0.27)	−0.01 (−0.76 to 0.73)
Birth weight-for-age Z-scores						
Hb: 80–89 g/L	−1.27±0.99	−1.06±1.11	−1.48±1.90	0.20 (−0.96 to 1.24)	−0.22 (−3.61 to 3.37)	0.42 (−2.12 to 0.56)
Hb: 90–99 g/L	−0.98±1.07	−1.32±1.06	−1.49±1.07	**0.50 (0.11 to 0.89**)	*0.36 (−0.04 to 0.86)*	**0.14 (0.05 to 0.22**)
Hb: 100–109 g/L	−1.12±1.11	−1.25±1.09	−1.45±0.98	**0.33 (0.24 to 0.42**)	0.14 (−0.14 to 0.41)	0.19 (−0.07 to 0.40)
Hb: 110–119 g/L	−1.23±1.06	−1.09±0.88	−1.15±1.01	−0.07 (−0.35 to 0.20)	−0.13 (−0.30 to 0.05)	0.08 (−0.20 to 0.36)
Hb: 120–129 g/L	−1.20±1.03	−1.01±0.99	−0.98±0.97	−0.21 (−0.58 to 0.17)	−0.19 (−0.46 to 0.06)	−0.02 (−0.36 to 0.14)
Hb≥130 g/L	−1.22±1.06	−1.12±0.88	−0.95±0.88	−0.22 (−0.54 to 0.09)	−0.10 (−0.43 to 0.23)	−0.13 (−0.64 to 0.38)
Birth head circumference-for-age Z-scores						
Hb: 80–89 g/L	−1.07±1.00	−0.81±1.25	−1.19±1.08	0.12 (−0.25 to 0.49)	−0.21 (−2.77 to 2.35)	0.32 (−2.16 to 2.79)
Hb: 90–99 g/L	−0.87±1.23	−1.09±1.18	−1.13±1.17	**0.26 (0.02 to 0.51**)	*0.20 (−0.03 to 0.43)*	0.06 (−0.31 to 0.42)
Hb: 100–109 g/L	−0.93±1.10	−0.93±1.07	−1.13±1.18	0.20 (−0.13 to 0.54)	−0.01 (−0.20 to 0.11)	0.20 (−0.10 to 0.48)
Hb: 110–119 g/L	−0.86±1.16	−0.71±1.00	−0.62±1.09	**−0.25 (−0.34 to 0.15**)	−0.11 (−0.46 to 0.24)	−0.01 (−0.21 to 0.20)
Hb: 120–129 g/L	−0.87±1.17	−0.67±1.08	−0.54±1.18	−0.32 (−0.65 to 0.10)	−0.23 (−0.62 to 0.15)	−0.08 (−0.45 to 0.30)
Hb≥130 g/L	−0.75±1.07	−0.70±1.06	−0.53±1.01	−0.20 (−0.55 to 0.17)	−0.05 (−0.45 to 0.56)	−0.16 (−0.22 to 0.34)

Sample size (n) for each Hb category; Hb: 80–89 g/L (168); Hb: 90–99 g/L (305); Hb: 100–109 g/L (444); Hb: 110–119 g/L (453); Hb: 120–129 g/L (427); and Hb ≥130 g/L (646); Arm 1: received nutrition supplement before and during pregnancy (preconception to delivery); Arm 2: nutrition supplement only during pregnancy (from ~12 weeks of gestation to delivery); Arm 3: no nutrition supplement (control Arm).

* Using meta-analytic approach, pooled mean differences in Z-scores for length, weight, and head circumference-for-age were calculated by pooling site-specific results of effect measure modification from Pakistan, India, Democratic Republic of Congo (DRC) and Guatemala (online supplemental Table 4) using WHO child growth standards (non-gestational age adjusted data); The effect sizes in bold are statistically significant (p<0·05) and effect sizes in italic are marginally significant (p~0·06).

Hb, haemoglobin.

The comparison between Arm 1 and Arm 2 (SQ-LNS during pregnancy only) revealed that the pooled mean differences (95% CI) in the Z-scores for all anthropometric indicators did not significantly differ across any of the Hb categories (figure 2 and table 1). The Arm 2 versus Arm 3 comparison showed that for women in Arm 2 with Hb 90–99 g/L, the pooled mean differences (95% CI) in the Z-scores were significantly greater for birth weight (0.14 (0.05 to 0.22)) only. As for the other comparisons between Arm 1 and Arms 2 and 3, no differences in the adjusted mean anthropometric Z-scores were observed for the Hb categories (g/L) above 110 g/L (figure 2 and table 1).

#### GA-adjusted anthropometric data

GA-adjusted data were consistent with the unadjusted findings, showing significantly greater adjusted pooled mean differences in Z-scores for length for Arm 1 (preconception-to-birth SQ-LNS) versus Arm 3 (control) women with Hb 90–99 g/L and 100–109 g/L (online supplemental Table 5). Country-specific findings showed a similar pattern, with the greatest mean differences in Z-scores for three anthropometric indicators observed in Arm 1 versus Arm 3 among women with Hb 90–99 g/L (online supplemental Tables 6-8).

No significant differences were observed for any anthropometric measurements in Arm 2 (SQ-LNS during pregnancy only) versus Arm 3 comparisons across all Hb categories (online supplemental Table 5).

## Discussion

Our findings suggest that starting SQ-LNS preconception and continuing through pregnancy (Arm 1) had the most pronounced effects on fetal growth among women with moderate anaemia, while no significant effects were observed for those with mild anaemia or Hb concentrations ≥120 g/L. Compared with the control group (Arm 3), women with moderate anaemia in Arm 1 showed the greatest improvements in length-for-age Z-scores, with intermediate effects on birth weight and minimal impact on head circumference. In contrast, supplementation initiated at 12 weeks of pregnancy (Arm 2) had no or only modest impact on anthropometric outcomes across any Hb category.

The findings regarding the benefits of SQ-LNS before conception and during pregnancy for moderately anaemic women align with expectations based on nutritional physiology.^20 21^ Specifically, since anaemia is multifactorial,^22^ for women with lower Hb and likely iron and other nutrient deficiencies, improving nutritional status broadly through comprehensive supplementation theoretically may mitigate non-nutritional factors (eg, inflammation, oxidative stress) and lead to better birth outcomes.^23^ Characterisation of such potential interactions is beyond the scope of this analysis.

Interestingly, we did not observe a measurable benefit of preconception SQ-LNS among women with Hb 80–89 g/L, despite the hypothesis that women with lower Hb concentrations might derive greater advantage from nutritional supplementation. The lack of a measurable benefit of preconception SQ-LNS among women with Hb 80–89 g/L may reflect the small sample size within this subgroup, which was largely driven by participants from India and Pakistan, and the exclusion of women with Hb <80 g/L, truncating the lower range of Hb and limiting variability. Additionally, anaemia in this range may be influenced by non-nutritional factors, such as inflammation, haemoglobinopathies (eg, thalassemia, sickle cell disease), infectious conditions (malaria, tuberculosis, *Helicobacter pylori*, hookworm) and heavy menstrual blood loss,^24^,^27^ which may limit the potential for nutrient-based interventions to improve fetal growth. Therefore, the findings for women with Hb 80–89 g/L should be cautiously interpreted in light of these biological and methodological considerations.

Our findings indicate that neither mild anaemia (Hb 110–119 g/L) nor the absence of anaemia (Hb≥120 g/L) significantly modified the response to SQ-LNS supplementation. Fetal growth markers (length, weight and head circumference) did not differ across the three intervention arms for these Hb categories, suggesting that supplementation had limited additional impact in women with at least marginally adequate iron and nutrient status. This may reflect a potential ceiling effect, wherein the benefits of supplementation are more pronounced among women with moderate anaemia, possibly due to their greater baseline nutrient deficiencies. In contrast, women with mild anaemia or no anaemia may have had relatively sufficient micronutrient stores, limiting the extent to which additional supplementation could further improve fetal growth.

The findings of the current analysis build on prior findings,^1^ which demonstrated that the response of anaemic women to preconception nutritional supplementation was greater than that of non-anaemic women. The current study refines these observations by showing that the response of women with moderate (and possibly severe) anaemia prior to conception was significantly greater than mildly anaemic women in terms of birth size (especially length). These results suggest that the degree of maternal anaemia, perhaps as a proxy for more extensive nutritional risk, may influence the extent of benefit from nutritional interventions, highlighting a potential opportunity for more tailored nutritional strategies to support those at higher risk.

### Strengths and limitations

The strengths of the current analysis include its reliance on a robust, multi-country dataset, enabling the identification of women with specific Hb concentrations who may derive the greatest benefit from preconception nutrition supplements. By examining the effects of nutritional interventions on three distinct markers of fetal growth across a broad range of pre-pregnancy Hb concentrations, this study addresses a critical gap in the literature, as no prior research has comprehensively explored these associations. The inclusion of diverse populations enhances the generalisability of the findings, allowing for more informed, globally relevant recommendations. Furthermore, the detailed stratification of Hb concentrations allows for a more nuanced understanding of the relationship between maternal nutritional status and fetal growth markers, providing insights into individuals with the highest risk and potential for response to nutrition interventions. While this approach highlights the importance of considering targeted nutritional supplementation, further research is needed to fully explore its broader implications.

Nevertheless, the findings should also be interpreted with caution due to inherent limitations. First, we could not assess the effect of supplements on intrauterine growth among women with Hb < 80 g/L (4%) since they were not included in the WF trial. Second, the use of single-drop capillary blood samples analysed with the 201+HemoCue device could have introduced measurement errors. However, field teams were trained on appropriate methodology to prevent haemodilution from excessive interstitial fluid. Despite this, the known precision and accuracy issues associated with measuring Hb using single-drop capillary blood samples could have impacted the validity of the Hb measurements,^28 29^ particularly when analysing smaller subgroups based on Hb concentration. Third, the use of different Hb measurement methods across sites (Sahli’s method in India and HemoCue 201+in Pakistan, DRC and Guatemala) could affect the pooled findings by contributing to heterogeneity of results and to inconsistent classification of Hb categories. While HemoCue 201+employs a photometric technique with high analytic sensitivity and accuracy, the Sahli method relies on the visual comparison of acid hematin formation and is known to have lower precision due to its dependence on subjective colour matching, ambient lighting and potential degradation of colour standards.^30^ Consequently, Hb values from the India site may be subject to greater inter-observer variability and wider measurement error compared with values obtained using HemoCue. However, this limitation largely affects descriptive comparisons rather than inferential conclusions. To mitigate this, we conducted site-specific analyses in addition to pooled analyses; therefore, site-level results are not affected by cross-site differences in measurement technique. For the pooled analyses, each site used the same Hb measurement method consistently across all trial arms, meaning that misclassification is unlikely with respect to intervention arm. This reduces systematic bias in estimated intervention effects, although it may contribute to increased variability and attenuation of true associations. Lastly, our analysis was limited to assessing anaemia based only on Hb concentration at screening, measured using HemoCue on capillary blood. While this method remains common in field settings, recent evidence highlights the importance of incorporating additional biomarkers to assess iron status and inflammation to improve the accuracy of anaemia classification and to better differentiate iron deficiency from other causes of anaemia.^31^,^33^ The most recent evidence also favours venous blood sampling for Hb measurement to enhance reliability and comparability across studies.^28 29^ Although baseline data on iron deficiency, inflammation and genetic disorders were not available, we acknowledge significant variability in anaemia prevalence across study sites, with India having a much higher prevalence (92%) compared with Guatemala (14%). Nonetheless, site-specific results showed consistent findings with similar patterns of response to the intervention, as was true for earlier analysis that examined only presence or absence of anaemia based on Hb cut-off of 120 g/L.^1^

## Conclusion

The benefits of preconception SQ-LNS supplementation on intrauterine growth were most pronounced in women with pre-pregnancy moderate anaemia, whereas no benefits were observed in women with mild anaemia.

### Implications for policy, practice and future research

The findings of the current research study suggest potential advantages of prioritising subgroups with at least moderate anaemia for preconception nutrition interventions. Specifically, strategies designed to screen and target women with moderate and perhaps severe anaemia for nutrition supplementation may yield the greatest benefits for intrauterine growth. Screening and identifying high-risk women by measuring their pre-pregnancy Hb using point-of-care devices could be a pragmatic and relatively cost-effective approach. Before providing nutrition supplements in low- and middle-income countries, this may allow programme implementers to identify women with the maximum potential to respond to supplementation.

In many countries, multiple micronutrient supplements (MMSs) are increasingly promoted as an alternative to standard IFA, supported by evidence from recent meta-analyses showing modest but consistent improvements in birth outcomes, particularly higher birth weight and reduced risk of low birth weight, when MMS is compared with IFA.^34^,^3838^ Current WHO guidance does not endorse the universal replacement of IFA with MMS but allows its use under specific conditions, such as settings with high prevalence of micronutrient deficiencies and adequate capacity for monitoring and evaluation.^43^ In this evolving policy landscape, the potential added value of SQ-LNS may differ when MMS, rather than IFA, is the comparator. MMS and SQ-LNS differ in both composition and intended purpose: SQ-LNS provides not only a broader micronutrient profile but also energy and essential fatty acids not supplied by MMS. As countries increasingly adopt MMS, future research should directly compare SQ-LNS with the widely used UNIMMAP MMS formulation, evaluating both effectiveness and cost-effectiveness, particularly in food-insecure populations, to determine the relative advantages of each approach.

## Supplementary material

10.1136/bmjgh-2025-020160 online supplemental file 1

10.1136/bmjgh-2025-020160 online supplemental file 2

## Data Availability

Data are available in a public, open access repository.
