# Supplementary material for: Women with moderate anaemia prior to conception benefited most from nutrition interventions: a secondary analysis of the Women First preconception maternal nutrition trial
Source: BMJ Glob Health. 2026 Jan 23;11(1):e020160. doi: 10.1136/bmjgh-2025-020160 (PMC12853505; doi:10.1136/bmjgh-2025-020160)
Supplement: online supplemental file 1 [file bmjgh-11-1-s001.docx]

**Supplementary Material**

**Table of contents**

**Supplemental Figure 1**: Flow of study participants in Women First Preconception Maternal Nutrition Trial …..…2

**Supplemental Table 1**: Distribution of baseline sociodemographic characteristics by Site and intervention Arm (India and Pakistan) …………………………………………………………………………………………………...3

**Supplemental Table 2**: Distribution of baseline sociodemographic characteristics of women by Site and intervention Arm (DRC and Guatemala) ……………………………………………………………………………...4

**Supplemental Table 3:** Study participants' characteristics by pre-pregnancy hemoglobin categories……………….5

**Supplemental Table 4**: Adjusted mean differences in Z-scores for birth length, weight, and head circumference-for-age between randomized arms: Stratified by pre-pregnancy hemoglobin (g/L)-Site-specific analysis………………………………………………………………………………………………………………..7

**Supplemental Table 5**: Pooled mean difference (95% CIs) in Z-scores for birth length, weight, and head circumference-for-gestational age between the randomized arms: Stratified by pre-pregnancy hemoglobin (g/L) categories- (Gestational age adjusted data: INTERGROWTH-21st fetal growth standards, n =1456) …………….9

**Supplemental Table 6**: Adjusted mean differences in Z-scores for birth length, weight, and head circumference-for-gestational age between the randomized arms: Stratified by pre-pregnancy hemoglobin (g/L)- (Pakistan (n = 456) ……………………………………………………………………………………………………………………….10

**Supplemental Table 7**: Adjusted mean differences in Z-scores for birth length, weight, and head circumference-for-gestational age between randomized arms: Stratified by pre-pregnancy hemoglobin (g/L)-India (n = 507) …….11

**Supplemental Table 8**: Adjusted mean differences in Z-scores for length, weight, and head circumference-for-gestational age between randomized arms: Stratified by pre-pregnancy hemoglobin (g/L)- (Guatemala (n = 493) ……………………………………………………………………………………………………………………….12

Livebirths, n=836

Livebirths, n=885

Livebirths, n=865

Birth weight, length, and head circumference within 48 hours of birth, n=801

Birth weight, length, and head circumference within 48 hours of birth, n=837

Birth weight, length, and head circumference within 48 h of birth, n= 805

**Analytical sample: 2,443**

Ineligible (n=4,865)

n

Eligible for consent and randomization (n=7,686)

Declined to participate (n=299)

Arm 1

n=2,465

Arm 2

n=2,460

Arm 3

n=2,462

Became pregnant and were followed for birth outcomes, n=1,029

Became pregnant and were followed for birth outcomes, n=1,095

Became pregnant and were followed for birth outcomes, n=1,127

Birth outcome:

n=1,001 (women),

1,010 (newborns)

Birth outcome:

n=1,068 (women), 1,078 (newborns)

Birth outcome:

n=1,094 (women),

1,100 (newborns)

Miscarriages

/Stillbirths: 174

Miscarriages

/Stillbirths: n=215

Miscarriages

/Stillbirths:213

**Outcome**

**Enrollment and Randomization**

**Follow up**

**Screening**

Pre-pregnancy hemoglobin measured

Exited: 1,436

Exited: 1,333

Exited: 1,367

Exited: 28

Exited: 33

Exited: 27

Collected baseline data and randomized (n=7,387)

Women screened for eligibility, n=12,551

**Preconception Phase**

**Pregnancy and birth**

**Supplemental Figure 1:** Flow of study participants in Women First Preconception Maternal Nutrition Trial from India, Pakistan, the Democratic Republic of Congo (DRC), and Guatemala by Arm showing data available on maternal hemoglobin and outcome (birth weight, length, and head circumference) for the final analytical sample. Arm 1: Received small-quantity lipid-based nutrient supplement (SQ-LNS) before and during pregnancy (preconception to delivery); Arm 2: Received SQ-LNS only during pregnancy (from ~ 12 weeks of gestation to delivery); Arm 3: Received no nutrition supplement (control Arm). Details are previously published in the main paper of Women First Trial (4).

| **Supplemental Table 1: Distribution of baseline sociodemographic characteristics by Site and intervention Arm (India and Pakistan)** | | | | | | | | | |
| --- | --- | --- | --- | --- | --- | --- | --- | --- | --- |
|  | **India (n = 590)** | | | | | **Pakistan (n = 663)** | | | |
|  | ***Arm 1 (Preconception) (n=196)*** | ***Arm 2***  ***(Pregnancy)***  ***(n=200)*** | ***Arm 3 (Control)***  ***(n=194)*** | ***Total (n=590)*** | ***Arm 1 (Preconception)***  ***(n=237)*** | | ***Arm 2***  ***(Pregnancy)***  ***(n=223)*** | ***Arm 3***  ***(Control) (n=203)*** | ***Total (n=663)*** |
| **Age categories, *n (%)*** |  |  |  |  |  | |  |  |  |
| <20 Years | 46 (23·5) | 52 (26·0) | 49 (25·3) | 147 (24·9) | 43 (18·1) | | 45 (20·2) | 28 (23·8) | 116 (17·5) |
| ≥ 20 years | 150 (76·5) | 148 (74·0) | 145 (74·7) | 443 (75·1) | 194 (81·8) | | 178 (79·8) | 175 (86·2) | 547 (82·5) |
| **Parity, *n (%)*** |  |  |  |  |  | |  |  |  |
| 0 child | 60 (30·6) | 48 (24·0) | 43 (22·2) | 151 (25·6) | 74 (31·2) | | 60 (26·9) | 56 (27·6) | 190 (28·7) |
| ≥1 child | 136 (69·4) | 152 (76·0) | 151(77·8) | 439 (74·4) | 163 (68·8) | | 163 (73·1) | 147 (72·4) | 473 (71·3) |
| **Maternal Education, *n (%)*** |  |  |  |  |  | |  |  |  |
| No formal education | 16 (8·2) | 12 (6·0) | 17 (8·7) | 45 (7·6) | 207 (87·3) | | 181 (81·2) | 174 (85·7) | 562 (84·7) |
| Formal Education | 180 (91·8) | 188 (94·0) | 177 (91·3) | 545 (92·4) | 30 (12·7) | | 42 (18·8) | 29 (14·3) | 101 (15·3) |
| **Maternal BMI (kg/m^2^), *n (%)*** |  |  |  |  |  | |  |  |  |
| Underweight (<18·5) | 77 (39·3) | 78 (39·0) | 64 (32·9) | 219 (37·1) | 83 (35·0) | | 73 (32·7) | 79 (38·9) | 235 (35·4) |
| Normal weight (18·5-24·9) | 98 (50·0) | 112 (56·0) | 113 (58·2) | 323 (54·7) | 141 (59·5) | | 139 (62·3) | 116 (57·1) | 396 (59·7) |
| Overweight or obese (≥25) | 21 (10·7) | 10 (5·0) | 17 (8·7) | 48 (8·1) | 13 (5·5) | | 11 (4·9) | 8 (3·9) | 32 (4·8) |
| **Socioeconomic status**^1^**, n (%)** |  |  |  |  |  | |  |  |  |
| 0 indicator | 0 (0·0) | 0 (0·0) | 0 (0·0) | 0 (0·0) | 8 (3·4) | | 2 (0·9) | 9 (4·4) | 19 (2·8) |
| 1-2 indicators | 23 (11·7) | 20 (10·0) | 15 (7·7) | 58 (9·8) | 114 (48·1) | | 102 (45·7) | 86 (42·4) | 302 (45·5) |
| 3- 4 indicators | 118 (60·0) | 134 (67·0) | 123 (63·4) | 375 (63·6) | 79 (33·3) | | 85 (38·1) | 77 (37·9) | 241 (36·3) |
| 5-6 indicators | 157 (26·6) | 55 (28·1) | 46 (23·0) | 157 (26·6) | 36 (15·2) | | 34 (15·2) | 31 (15·3) | 101 (15·2) |
| **Pre-pregnancy Hb (g/L), mean ± SD** | 101±11 | 102±12 | 101±11 | 100±10 | 109±16 | | 109±15 | 109±16 | 109±16 |
| **Pre-pregnancy anemia (2), *n (%)*** |  |  |  |  |  | |  |  |  |
| Anemia (Hb <120 g/L) | 177 (90·3) | 185 (92·5) | 182 (93·8) | 544 (92·2) | 166 (70·0) | | 160 (71·7) | 146 (71·9) | 472 (71·2) |
| No anemia (Hb ≥120 g/L) | 19 (9·7) | 15 (7·5) | 12 (6·2) | 46 (7·8) | 71 (29·9) | | 63 (28·2) | 57 (28·1) | 191 (28·8) |
| **Anemia (WHO classification) (2), n *(%)*** |  |  |  |  |  | |  |  |  |
| No anemia (Hb ≥120 g/L) | 19 (9·7) | 15 (7·5) | 12 (6·2) | 46 (7·8) | 71 (29·9) | | 63 (28·2) | 57 (28·1) | 191 (28·8) |
| Mild anemia (Hb:110-119 g/L) | 36 (18·4) | 35 (17·5) | 23 (11·8) | 94 (15·9) | 45 (18·9) | | 49 (21·9) | 40 (20·0) | 134 (20·2) |
| Moderate anemia (Hb: 80-109 g/L) | 141 (71·8) | 150 (75·0) | 159 (81·9) | 450 (76·2) | 121 (51·0) | | 111 (49·7) | 106 (50·7) | 338 (50·9) |
| **Hemoglobin categories (g/L), *n (%)*** |  |  |  |  |  | |  |  |  |
| Hb: 80-89 | 25 (12·7) | 25 (12·5) | 24 (12·4) | 74 (12·5) | 32 (13·5) | | 22 (9·8) | 30 (14·8) | 84 (12·7) |
| Hb: 90-99 | 53 (27·0) | 57 (28·5) | 54 (27·8) | 164 (27·8) | 41 (17·3) | | 35 (15·7) | 35 (15·7) | 111 (16·7) |
| Hb: 100-109 | 63 (32·14) | 68 (34·0) | 81 (41·7) | 212 (35·9) | 48 (20·2) | | 54 (24·2) | 41 (20·2) | 143 (21·6) |
| Hb: 110-119 | 36 (18·4) | 35 (17·5) | 23 (11·8) | 94 (15·9) | 45 (18·9) | | 49 (21·9) | 40 (20·0) | 134 (20·2) |
| Hb: 120-129 | 17 (8·7) | 14 (7·0) | 9 (4·64) | 40 (6·8) | 43 (18·1) | | 41 (18·4) | 37 (18·2) | 121 (18·2) |
| Hb ≥130 | 2 (1·02) | 1 (0·5) | 3 (1·55) | 6 (1·0) | 28 (11·8) | | 22 (9·8) | 20 (9·9) | 70 (10·6) |

*BMI: Body mass index; Hb: Hemoglobin (g/L); SD: Standard deviation; WHO: World Health Organization.*

*Arm 1: Received small-quantity lipid-based nutrient supplement (SQ-LNS) before and during pregnancy (preconception to delivery); Arm 2: Received SQ-LNS only during pregnancy (from ~ 12 weeks of gestation to delivery); Arm 3: Received no nutrition supplement (control Arm)*

^1^ *The tally for socioeconomic status is based on the list of six indicators including electricity, sanitation, improved water supply, man-made flooring, improved water source, improved cooking fuels, and household assets.*

*No significant differences were found in baseline characteristics across three Arms for the two sites.*

| **Supplemental Table 2: Distribution of baseline sociodemographic characteristics of women by Site and intervention Arm (DRC and Guatemala)** | | | | | | | | |
| --- | --- | --- | --- | --- | --- | --- | --- | --- |
|  | **The Democratic Republic of Congo (n = 577)** | | | | **Guatemala (n = 613)** | | | |
|  | ***Arm 1 (Preconception) (n=181)*** | ***Arm 2***  ***(Pregnancy)***  ***(n=197)*** | ***Arm 3***  ***(Control) (n=199)*** | ***Total (n=577)*** | ***Arm 1 (Preconception) (n=187)*** | ***Arm 2***  ***(Pregnancy)***  ***(n=217)*** | ***Arm 3***  ***(Control) (n=209)*** | ***Total (n=613)*** |
| **Age categories, *n (%)*** |  |  |  |  |  |  |  |  |
| <20 Years | 40 (22·1) | 55 (27·9) | 46 (23·1) | 141 (24·4) | 24 (12·8) | 32 (14·7) | 34 (16·3) | 90 (14·7) |
| ≥ 20 years | 141 (77·9) | 142 (72·1) | 153 (76·8) | 436 (75·6) | 163 (87·2) | 185 (85·2) | 175 (83·7) | 523 (85·3) |
| **Parity, *n (%)*** |  |  |  |  |  |  |  |  |
| 0 child | 38 (20·9) | 44 (22·3) | 39 (19·6) | 121 (20·9) | 14 (7·5) | 13 (5·9) | 8 (3·8) | 35 (5·7) |
| ≥1 child | 143 (79·1) | 153(77·7) | 160 (80·4) | 456 (79·1) | 173 (92·5) | 204 (94·1) | 201 (96·2) | 578 (94·3) |
| **Maternal Education, *n (%)*** |  |  |  |  |  |  |  |  |
| No formal education | 49 (27·1) | 45 (22·8) | 41 (20·6) | 135 (23·4) | 14 (7·5) | 14 (6·4) | 21(10·0) | 49 (7·9) |
| Formal education | 132 (72·9) | 152 (77·2) | 158 (79·4) | 442 (76·6) | 173 (92·5) | 203 (93·6) | 188 (90·0) | 564 (92·1) |
| **Maternal BMI (kg/m^2^), *n (%)*** |  |  |  |  |  |  |  |  |
| Underweight (<18.5) | 26 (14·4) | 41 (20·8) | 34 (17·1) | 101 (17·5) | 2 (1·1) | 5 (2·3) | 1 (0·5) | 8 (1·3) |
| Normal weight (18·5-24·9) | 144 (79·5) | 150 (76·1) | 154 (77·4) | 448 (77·6) | 93 (49·7) | 106 (48·8) | 113 (54·1) | 312 (50·9) |
| Overweight or obese (≥25) | 11 (6·1) | 6 (3·0) | 11 (5·5) | 28 (4·8) | 92 (49·2) | 106 (48·8) | 95 (45·4) | 293 (47·8) |
| **Socioeconomic status**^1^**, n (%)** |  |  |  |  |  |  |  |  |
| 0 indicator | 99 (54·7) | 109 (55·3) | 101 (50·7) | 309 (53·5) | 0 (0·0) | 0 (0·0) | 0 (0·0) | 0 (0·0) |
| 1-2 indicators | 78 (43·1) | 84 (42·6) | 98 (49·2) | 260 (45·1) | 24 (12·8) | 29 (13·4) | 20 (9·6) | 73 (11·9) |
| 3- 4 indicators | 4 (2·2) | 4 (2·0) | 0 (0) | 8 (1·4) | 112 (59·9) | 122 (56·2) | 132 (63·2) | 366 (59·7) |
| 5-6 indicators | 0 (0·0) | 0 (0·0) | 0 (0·0) | 0 (0·0) | 51 (27·3) | 66 (30·4) | 57 (27·3) | 174 (28·4) |
| **Pre-pregnancy Hb (g/L), mean ± SD** | 121±13 | 121±11 | 121±13 | 121±12 | 140±14 | 138±14 | 136±11 | 138±13 |
| **Pre-pregnancy anemia (2), *n (%)*** |  |  |  |  |  |  |  |  |
| Anemia (Hb <120 g/L) | 88 (48·6) | 86 (43·6) | 91 (45·7) | 265 (45·9) | 28 (14·9) | 33 (15·2) | 28 (13·4) | 89 (14·5) |
| No anemia (Hb ≥120 g/L) | 93 (51·4) | 111 (56·3) | 108 (54·2) | 312 (54·1) | 159 (85·0) | 184 (84·8) | 181 (86·6) | 524 (85·5) |
| **Anemia (WHO classification) (2), n *(%)*** |  |  |  |  |  |  |  |  |
| No anemia (Hb ≥120 g/L) | 93 (51·4) | 111 (56·3) | 108 (54·2) | 312 (54·1) | 159 (85·0) | 184 (84·8) | 181 (86·6) | 524 (85·5) |
| Mild anemia (Hb:110-119 g/L) | 55 (30·4) | 61 (30·9) | 56 (28·1) | 172 (29·8) | 18 (9·6) | 17 (7·8) | 18 (8·6) | 53 (8·6) |
| Moderate anemia (Hb: 80-109 g/L) | 33 (18·2) | 25 (12·7) | 35 (17·6) | 93 (16·1) | 10 (5·3) | 16 (7·3) | 10 (4·74) | 36 (5·8) |
| **Hemoglobin categories (g/L), *n (%)*** |  |  |  |  |  |  |  |  |
| Hb: 80-89 | 1 (0·55) | 0 (0·0) | 1 (0·5) | 2 (0·3) | 1 (0·52) | 4 (1·8) | 3(1·44) | 8 (1·3) |
| Hb: 90-99 | 8 (4·4) | 3 (1·5) | 8 (4·0) | 19 (3·3) | 2 (1·1) | 5 (2·3) | 4 (1·9) | 11 (1·8) |
| Hb: 100-109 | 24 (13·3) | 22 (11·2) | 26 (13·1) | 72 (12·5) | 7 (3·7) | 7 (3·2) | 3 (1·4) | 17 (2·8) |
| Hb: 110-119 | 55 (30·4) | 61 (30·9) | 56 (28·1) | 172 (29·8) | 18 (9·6) | 17 (7·8) | 18 (8·6) | 53 (8·6) |
| Hb: 120-129 | 47 (25·9) | 67 (34·0) | 43 (21·6) | 157 (27·2) | 36 (19·2) | 31 (14·3) | 42 (20·1) | 109 (17·8) |
| Hb ≥130 | 46 (25·4) | 44 (22·3) | 65 (32·6) | 155 (26·8) | 123 (65·8) | 153 (70·5) | 139 (66·5) | 415 (67·7) |

*BMI: Body mass index; Hb: Hemoglobin (g/L); SD: Standard deviation; WHO: World Health Organization.*

*Arm 1: Received small-quantity lipid-based nutrient supplement (SQ-LNS) before and during pregnancy (preconception to delivery); Arm 2: Received SQ-LNS only during pregnancy (from ~ 12 weeks of gestation to delivery); Arm 3: Received no nutrition supplement (control Arm)*

^1^ *The tally for socioeconomic status is based on the list of six indicators including electricity, sanitation, improved water supply, man-made flooring, improved water source, improved cooking fuels, and household assets.*

*No significant differences were found in baseline characteristics across three Arms for the two sites.*

| **Supplemental Table 3: Study participants' characteristics by pre-pregnancy hemoglobin categories** | | | | | | |
| --- | --- | --- | --- | --- | --- | --- |
| **Maternal characteristics** | **Pre-pregnancy hemoglobin (g/L) categories** | | | | | |
|  | **Hb**  **80-89** | **Hb**  **90-99** | **Hb**  **100-109** | **Hb**  **110-119** | **Hb**  **120-129** | **Hb**  **≥130** |
| ***All sites combined (India, Pakistan, DRC, and Guatemala) n=2,443*** | | | | | | |
| **Age categories, *n (%)*** | | | | | | |
| <20 Years | 42 (25) | 58 (19.0) | 75 (16.9) | 108 (23.8) | 98 (23.0) | 113 (17.5) |
| ≥ 20 Years | 126 (75) | 247 (81.0) | 369 (83.1) | 345(76.2) | 329 (77.0) | 533 (82.5) |
| **Parity, *n (%)*** | | | | | | |
| Nulliparous | 46 (27.4) | 61 (20.0) | 96 (21.6) | 101 (22.3) | 102 (23.9) | 91 (14.1) |
| At least one child | 122 (72.6) | 244 (80.0) | 348 (78.4) | 352 (77.7) | 325 (76.1) | 555 (85.9) |
| **Maternal Education, *n (%)*** | | | | | | |
| No formal education | 88 (52.4) | 113 (37.0) | 159 (35.8) | 168 (37.1) | 148 (34.6) | 115 (17.8) |
| Formal education | 80 (47.6) | 192 (62.9) | 285 (64.2) | 285 (62.9) | 279 (65.3) | 531 (82.2) |
| **Maternal BMI (kg/m^2^) categories, *n (%)*** | | | | | | |
| Underweight (<18.5) | 55 (32.7) | 99 (32.5) | 146 (32.8) | 117 (25.8) | 88 (20.6) | 58 (9.0) |
| Normal weight (18.5-24.9) | 104 (61.9) | 185 (60.7) | 264 (59.5) | 293 (64.7) | 265 (62.1) | 368 (57.0) |
| Overweight or obese (≥25) | 9 (5.4) | 21 (7.0) | 34 (7.7) | 43 (9.5) | 74 (17.3) | 220 (34.1) |
| **Socioeconomic status, n (%)** | | | | | | |
| low SES (0-2 indicators) | 107 (64.0) | 211 (69.2) | 284 (64.0) | 209 (46.1) | 200 (46.8) | 411 (63.6) |
| High SES (3-6 indicators) | 61 (36.3) | 94 (30.8) | 160 (36.0) | 244 (53.8) | 227 (53.2) | 235 (36.4) |
| ***India (n=590)*** | | | | | | |
| **Age categories, *n (%)*** | | | | | | |
| <20 Years | 27 (36.5) | 35 (21.3) | 44 (20.7) | 26 (27.6) | 14 (35.0) | 1 (16.7) |
| ≥ 20 Years | 47 (63.5) | 129 (78.7) | 168 (79.2) | 68 (72.3) | 26 (65.0) | 5(83.3) |
| **Parity, *n (%)*** | | | | | | |
| Nulliparous | 24 (32.4) | 35 (21.3) | 53 (25.0) | 26 (27.7) | 11 (27.5) | 2 (33.3) |
| At least one child | 50 (67.6) | 129 (78.7) | 159 (75.0) | 68 (72.3) | 29 (72.5) | 4 (66.7) |
| **Maternal Education, *n (%)*** | | | | | | |
| No formal education | 11 (14.9) | 11 (6.71) | 16 (7.5) | 5(5.3) | 2(5.0) | 0 (0) |
| Formal education | 63 (85.1) | 153 (93.3) | 196 (92.4) | 89 (94.7) | 38 (95.0) | 6 (100) |
| **Maternal BMI (kg/m^2^) categories, *n (%)*** | | | | | | |
| Underweight (<18.5) | 30 (40.5) | 60 (36.6) | 76 (35.8) | 33 (35.1) | 18 (45.0) | 2 (33.3) |
| Normal weight (18.5-24.9) | 40 (54.0) | 94 (57.3) | 117 (55.2) | 52 (55.3) | 17 (42.5) | 3 (50.0) |
| Overweight or obese (≥25) | 4 (5.0) | 10 (6.0) | 19 (8.9) | 9 (9.5) | 5 (12.5) | 1 (16.6) |
| **Socioeconomic status, n (%)** | | | | | | |
| low SES (0-2 indicators) | 11 (14.8) | 17 (10.4) | 16 (7.5) | 10 (10.6) | 4(10.0) | 0(0) |
| High SES (3-6 indicators) | 63 (85.1) | 147 (90.0) | 196 (92.4) | 84 (89.4) | 36 (90.0) | 6 (100) |
| ***Pakistan (n=663)*** | | | | | | |
| **Age categories, *n (%)*** | | | | | | |
| <20 Years | 14 (16.7) | 17 (15.3) | 17 (11.9) | 30 (22.4) | 22 (18.2) | 16 (22.8) |
| ≥ 20 Years | 70 (83.3) | 94 (84.6) | 126 (88.1) | 104 (77.6) | 99 (81.8) | 54 (77.1) |
| **Parity, *n (%)*** | | | | | | |
| Nulliparous | 21 (25.0) | 20 (18.0) | 31 (21.7) | 39 (29.1) | 40 (33.1) | 39 (55.7) |
| At least one child | 63 (75.0) | 91 (82.0) | 112 (78.3) | 95 (71.0) | 81 (66.9) | 31 (44.3) |
| **Maternal Education, *n (%)*** | | | | | | |
| No formal education | 74 (88.1) | 94 (84.7) | 119 (83.2) | 117 (87.3) | 105 (86.8) | 53 (75.7) |
| Formal education | 10 (11.9) | 17 (15.3) | 24 (16.8) | 17 (12.7) | 16 (13.2) | 17 (24.3) |
| **Maternal BMI (kg/m^2^) categories, *n (%)*** | | | | | | |
| Underweight (<18.5) | 23 (27.4) | 36 (32.4) | 54 (37.8) | 50 (37.3) | 46 (38.0) | 26 (37.1) |
| Normal weight (18.5-24.9) | 59 (70.2) | 70 (63.1) | 84 (58.7) | 78 (58.2) | 65 (53.7) | 40 (57.1) |
| Overweight or obese (≥25) | 2 (2.4) | 5 (4.5) | 5(3.5) | 6(4.5) | 10(8.3) | 4 (5.7) |
| **Socioeconomic status, n (%)** | | | | | | |
| low SES (0-2 indicators) | 45 (53.6) | 54 (48.6) | 70 (48.9) | 56 (41.8) | 58 (47.9) | 38 (54.3) |
| High SES (3-6 indicators) | 39 (46.4) | 57 (51.3) | 73 (51.0) | 78 (58.2) | 63 (52.1) | 32 (45.7) |
| ***DRC (n=577)*** | | | | | | |
| **Age categories, *n (%)*** | | | | | | |
| <20 Years | 0 (0) | 6 (31.6) | 10 (13.9) | 42 (24.4) | 48 (24.4) | 35 (22.6) |
| ≥ 20 Years | 2 (100) | 13 (68.4) | 62 (86.1) | 130 (75.6) | 109 (69.4) | 120 (72.4) |
| **Parity, *n (%)*** | | | | | | |
| Nulliparous | 1 (50) | 5 (26.3) | 12 (16.7) | 34 (19.8) | 40 (25.5) | 29 (18.7) |
| At least one child | 1 (50) | 14 (73.7) | 60 (83.3) | 138 (80.2) | 117 (74.5) | 126 (81.3) |
| **Maternal Education, *n (%)*** | | | | | | |
| No formal education | 1 (50) | 7 (36.8) | 22 (30.5) | 42 (24.4) | 35 (22.3) | 28 (18.1) |
| Formal education | 1 (50) | 12 (63.2) | 50 (69.4) | 130 (75.6) | 122 (77.7) | 127 (81.9) |
| **Maternal BMI (kg/m^2^) categories, *n (%)*** | | | | | | |
| Underweight (<18.5) | 1 (50) | 3 (15.9) | 16 (22.2) | 34 (19.8) | 21 (13.4) | 26 (16.8) |
| Normal weight (18.5-24.9) | 1 (50) | 16 (84.2) | 54 (75.0) | 128 (74.4) | 129 (82.2) | 120 (77.4) |
| Overweight or obese (≥25) | 0(0) | 0(0) | 2(2.8) | 10 (5.8) | 7 (4.5) | 9 (5.8) |
| **Socioeconomic status, n (%)** | | | | | | |
| low SES (0-2 indicators) | 2 (100) | 19 (100) | 72 (100) | 171 (99.4) | 153 (97.4) | 152 (98.1) |
| High SES (3-6 indicators) | 0(0) | 0(0) | 0(0) | 1 (0.6) | 4 (2.5) | 3 (1.9) |
| ***Guatemala (n=613)*** | | | | | | |
| **Age categories, *n (%)*** | | | | | | |
| <20 Years | 1 (12.5) | 0(0) | 4 (23.5) | 10 (18.8) | 14 (12.8) | 61 (14.7) |
| ≥ 20 Years | 7 (87.5) | 11 (100) | 13 (76.5) | 43 (81.1) | 95 (87.2) | 354 (85.3) |
| **Parity, *n (%)*** | | | | | | |
| Nulliparous | 0(0) | 1 (9.1) | 0(0) | 2(3.8) | 11 (10.0) | 21 (5.1) |
| At least one child | 8 (100) | 10 (90.9) | 17 (100) | 51 (96.2) | 98 (90.0) | 394 (94.9) |
| **Maternal Education, *n (%)*** | | | | | | |
| No formal education | 6 (75) | 10 (90.9) | 15 (88.2) | 49 (92.4) | 103 (94.5) | 381 (91.8) |
| Formal education | 2 (25.0) | 1(9.1) | 2(11.8) | 4(7.5) | 6(5.5) | 34(8.2) |
| **Maternal BMI (kg/m^2^) categories, *n (%)*** | | | | | | |
| Underweight (<18.5) | 1(12.5) | 0(0) | 0(0) | 0(0) | 3(2.7) | 4(0.9) |
| Normal weight (18.5-24.9) | 4(50.0) | 5(45.4) | 9(52.9) | 35(66.0) | 54 (49.5) | 205 (49.4) |
| Overweight or obese (≥25) | 3(37.5) | 6(54.5) | 8(47.1) | 18(33.9) | 52(47.7) | 206(49.6) |
| **Socioeconomic status, n (%)** | | | | | | |
| low SES (0-2 indicators) | 3(37.5) | 4(36.4) | 2(11.7) | 7(13.2) | 12(11.0) | 45(10.8) |
| High SES (3-6 indicators) | 5(62.5) | 7(63.6) | 15(88.2) | 46 (86.8) | 97(88.9) | 370 (89.2) |

*Hb: Hemoglobin; BMI: Body mass index; The socio-economic status (SES) score is based on the presence of indicators, including electricity, access to an improved water source, sanitation, durable flooring, use of improved cooking fuels, and household assets. A low SES is defined as having two or fewer of these indicators, as outlined in a previously published Women's First (WF) study (1).*

**Supplemental Table 4: Adjusted mean differences in Z-scores for birth length, weight, and head circumference-for-age between randomized arms: Stratified by pre-pregnancy hemoglobin (g/L)-Site-specific results**

| **Three markers of intrauterine growth** | | ***Adjusted mean differences in Z-scores***^1^  ***(95% CIs)*** | | | | | | | | ***Adjusted mean differences in Z-scores***^1^  ***(95% CIs)*** | | | | | | |
| --- | --- | --- | --- | --- | --- | --- | --- | --- | --- | --- | --- | --- | --- | --- | --- | --- |
|  |  | ***Arm 1 vs. Arm 3*** | | ***Arm 1 vs. Arm 2*** | | | | ***Arm 2 vs. Arm 3*** | | ***Arm 1 vs. Arm 3*** | | ***Arm 1 vs. Arm 2*** | | | ***Arm 2 vs. Arm 3*** | |
|  | |  | | ***India (n = 590)*** | | | |  | |  | | ***Pakistan (n = 663)*** | | |  | |
| **Birth length-for-age Z scores** | | | | | | | | | |  | | | | | | |
| Hb: 80-89g/L | | 0.06 (-0.28, 0.41) | | | **-0.55 (-0.85, -0.25)**^2^ | **0.61 (0.38, 0.84)**^2^ | | | | 0.48 (-0.06, 1.01) | | | 0.01 (-0.57, 0.65) | | 0.47 (-0.02, 0.90) | |
| Hb: 90-99 g/L | | 0.32 (-0.08, 0.73) | | | 0.25 (-0.08, 0.57) | 0.07 (-0.09, 0.24) | | | | **0.73 (0.41, 1.04)**^2^ | | | 0.42 (-0.72, 1.41) | | 0.31 (-0.67, 1.14) | |
| Hb: 100-109 g/L | | 0.20 (-0.15, 0.54) | | | -0.03 (-0.34, 0.27) | **0.23 (0.02, 0.45)**^3^ | | | | 0.50 (-0.17, 1.16) | | | -0.13 (-0.72, 0.44) | | **0.63 (0.09, 1.17)**^3^ | |
| Hb: 110-119 g/L | | 0.14 (-0.51, 0.79) | | | 0.15 (-0.41, 0.72) | -0.01 (-0.60, 0.57) | | | | -0.20 (-0.39, 0.01) | | | -0.02 (-0.40, 0.37) | | -0.18 (-0.56, 0.20) | |
| Hb: 120-129 g/L | | -0.17 (-1.21, 0.08) | | | 0.02 (-0.56, 0.61) | -0.19 (-1.01, 0.63) | | | | 0.10 (-0.45, 0.66) | | | 0.04 (-0.44, 0.52) | | 0.06 (-0.51, 0.64) | |
| Hb ≥130 g/L | | Not defined^4^ | | | Not defined^4^ | Not defined^4^ | | | | -0.22 (-1.28, 0.84) | | | 0.02 (-0.52, 0.57) | | -0.24 (-1.19, 0.71) | |
| **Birth weight-for-age Z scores** | | | | | | | | | |  | | | | | | |
| Hb: 80-89g/L | | 0.03 (-0.28, 0.34) | | | **-0.40 (-0.72, -0.07)**^3^ | **0.42 (0.12, 0.73)**^3^ | | | | **0.43 (0.01, 0.86)**^3^ | | | -0.05 (-0.31, 1.1) | | 0.48 (-0.5, 0.57) | |
| Hb: 90-99 g/L | | 0.35 (-0.08, 0.78) | | | 0.22 (-0.16, 0.60) | 0.13 (-0.05, 0.31) | | | | **0.51 (0.13, 0.85)**^3^ | | | 0.28 (-0.30, 0.86) | | 0.23 (-0.34, 0.80) | |
| Hb: 100-109 g/L | | 0.28 (-0.07, 0.64) | | | 0.06 (-0.32, 0.43) | 0.22 (-0.04, 0.49) | | | | 0.40 (-0.10, 0.88) | | | 0.02 (-0.34, 0.35) | | 0.38 (-0.01, 0.78) | |
| Hb: 110-119 g/L | | 0.04 (-0.67, 0.75) | | | 0.14 (-0.55, 0.83) | -0.10 (-0.67, 0.47) | | | | **-0.25 (-0.49, -0.01)**^3^ | | | -0.16 (-0.64, 0.32) | | -0.10 (-0.43, 0.25) | |
| Hb: 120-129 g/L | | -0.67 (-1.73, 0.39) | | | -0.22 (-0.56, 0.12) | -0.45 (-1.43, 0.53) | | | | -0.21 (-0.71, 0.27) | | | -0.26 (-0.71, 0.20) | | 0.04 (-0.24, 0.33) | |
| Hb ≥130 g/L | | Not defined^4^ | | | Not defined^4^ | Not defined^4^ | | | | -0.18 (-0.75, 0.39) | | | 0.10 (-0.62, 0.82) | | -0.28 (-0.75, 0.39) | |
| **Birth head circumference-for-age Z scores** | | | | | | | | | |  | | | | | | |
| Hb: 80-89g/L | | 0.16 (-0.44, 0.75) | | | -0.41 (-0.85, 0.04) | 0.56 (-0.01, 1.13) | | | | 0.07 (-0.78, 0.84) | | | -0.04 (-0.66, 0.86) | | 0.11 (-0.63, 0.89) | |
| Hb: 90-99 g/L | | 0.25 (-0.05, 0.55) | | | 0.23 (-0.07, 0.53) | 0.02 (-0.23, 0.26) | | | | 0.39 (-0.12, 0.89) | | | 0.52 (-0.30, 1.33) | | -0.13 (-0.92, 0.66) | |
| Hb: 100-109 g/L | | 0.01 (-0.48, 0.51) | | | -0.04 (-0.43, 0.34) | 0.05 (-0.25, 0.36) | | | | 0.37 (-0.10, 0.84) | | | -0.03 (-0.34, 0.27) | | **0.42 (0.04, 0.80)**^3^ | |
| Hb: 110-119 g/L | | 0.21 (-0.71, 1.13) | | | 0.28 (-0.27, 0.83) | -0.07 (-0.67, 0.53) | | | | -0.30 (-0.57, 0.001) | | | -0.13 (-0.56, 0.30) | | -0.15 (-0.58, 0.27) | |
| Hb: 120-129 g/L | | -0.78 (-1.70, 0.15) | | | -0.20 (-0.64, 0.38) | -0.57 (-1.43, 0.28) | | | | -0.20 (-0.68, 0.29) | | | -0.32 (-0.73, 0.08) | | 0.12 (-0.29, 0.54) | |
| Hb ≥130 g/L | | Not defined^4^ | | | Not defined^4^ | Not defined^4^ | | | | -0.13 (-0.54, 0.30) | | | 0.18 (-0.30, 0.73) | | -0.31 (-0.96, 0.34) | |
|  | ***The Democratic Republic of Congo (n = 577)*** | | | | | | | |  | | ***Guatemala (n = 613)*** | | |  | |  |
| **Birth length-for-age Z scores** | | | | | | | | |  | | | | | | |  |
| Hb: 80-89g/L | Not defined^5^ | | Not defined^5^ | | | | Not defined^4^ | | **-1.95 (-2.16, -1.74)**^2^ | | **-1.43 (-2.57, -0.29)** ^3^ | | | -0.52 (-1.49, 0.45) | |  |
| Hb: 90-99 g/L | **0.85 (0.20, 1.49)**^3^ | | **0.77 (0.26, 1.28)**^3^ | | | | 0.07 (-0.81, 0.96) | | **2.32 (1.74, 2.91)**^2^ | | 0.63 (-0.41, 1.41) | | | **1.69 (0.97, 2.41)**^2^ | |  |
| Hb: 100-109 g/L | **0.70 (0.02, 1.36)**^3^ | | 0.60 (-0.02, 1.21) | | | | 0.10 (-0.40, 0.61) | | 0.40 (-1.01, 1.27) | | 0.56 (-0.31, 1.82) | | | **-0.16 (-1.22, -0.01)**^3^ | |  |
| Hb: 110-119 g/L | 0.22 (-0.22, 0.67) | | -0.08 (-0.50, 0.34) | | | | 0.29 (-0.04, 0.65) | | -0.20 (-0.99, 0.49) | | -0.42 (-1.17, 0.33) | | | 0.22 (-0.25, 0.70) | |  |
| Hb: 120-129 g/L | 0.07 (-0.34, 0.50) | | 0.24 (-0.23, 0.81) | | | | -0.16 (-0.61, 0.28) | | -0.44 (-0.97, 0.20) | | -0.43 (-0.86, 0.01) | | | -0.01 (-0.29, 0.27) | |  |
| Hb ≥130 g/L | 0.09 (-0.70, 0.87) | | -0.25 (-0.82, 0.32) | | | | 0.34 (-0.17, 0.86) | | -0.21 (-0.76, 0.33) | | **-0.04 (-0.47, -0.13)** ^3^ | | | -0.17 (0.48, 0.14) | |  |
| **Birth weight-for-age Z scores** | | | | | | | | |  | | | | | | |  |
| Hb: 80-89g/L | Not defined^5^ | | Not defined^5^ | | | | Not defined^5^ | | -0.68 (-1.56, 0.20) | | -0.46 (-1.06, 0.14) | | | -0.22 (-1.41, 0.95) | |  |
| Hb: 90-99 g/L | **0.70 (0.20, 1.21)**^3^ | | **0.54 (0.34, 0.74)**^2^ | | | | 0.16 (-0.43, 0.74) | | **2.35 (1.45, 3.34)**^2^ | | **0.89 (0.43, 1.47)**^2^ | | | **1.46 (0.83, 2.05)**^2^ | |  |
| Hb: 100-109 g/L | 0.34 (-0.08, 0.77) | | 0.35 (-0.05, 0.74) | | | | -0.002 (-0.36, 0.35) | | 0.40 (-0.56, 1.35) | | 0.32 (-0.54, 1.19) | | | 0.07 (-0.75, 0.90) | |  |
| Hb: 110-119 g/L | 0.09 (-0.18, 0.38) | | -0.14 (-0.44, 0.16) | | | | 0.23 (-0.10, 0.57) | | -0.04 (-0.52, 0.44) | | -0.23 (-0.84, 0.38) | | | 0.18 (-0.15, 0.51) | |  |
| Hb: 120-129 g/L | 0.05 (-0.40, 0.50) | | 0.11 (-0.41, 0.64) | | | | -0.06 (-0.47, 0.35) | | -0.37 (-0.81, 0.07) | | -0.33 (-0.75, 0.10) | | | -0.02 (-0.33, 0.28) | |  |
| Hb ≥130 g/L | -0.12 (-0.61, 0.37) | | -0.20 (-0.67, 0.28) | | | | 0.07 (-0.28, 0.43) | | -0.36 (-0.86, 0.14) | | **-0.09 (-0.40, -0.05)**^3^ | | | -0.27 (-0.63, 0.08) | |  |
| **Birth head circumference-for-age Z scores** | | | | | | | | |  | | | | | | |  |
| Hb: 80-89g/L | Not defined^5^ | | Not defined^5^ | | | | Not defined^5^ | | -0.26 (-0.95, 0.42) | | -0.91 (-1.87, 0.04) | | | 0.64 (-0.68, 1.98) | |  |
| Hb: 90-99 g/L | 0.89 (-0.13, 2.20) | | 0.75 (-0.07, 1.85) | | | | 0.15 (-1.30, 1.60) | | 1.56 (-0.45, 3.57) | | 0.96 (-0.88, 2.80) | | | 0.60 (-0.32, 1.54) | |  |
| Hb: 100-109 g/L | 0.04 (-0.76, 0.83) | | -0.19 (-0.81, 0.43) | | | | 0.22 (-0.16, 0.33) | | 0.44 (-0.45, 1.32) | | 0.30 (-0.86, 1.46) | | | 0.14 (-0.40, 0.67) | |  |
| Hb: 110-119 g/L | **-0.25 (-0.35, -0.15)**^2^ | | -0.22 (-0.60, 0.14) | | | | -0.03 (-0.30, 0.24) | | -0.12 (-0.84, 0.61) | | -0.30 (-0.96, 0.35) | | | 0.18 (-0.20, 0.57) | |  |
| Hb: 120-129 g/L | -0.19 (-0.64, 0.26) | | 0.17 (-0.37, 0.73) | | | | -0.36 (-0.86, 0.13) | | -0.47 (-0.96, 0.02) | | -0.47 (-0.96, 0.98) | | | -0.02 (-0.33, 0.29) | |  |
| Hb ≥130 g/L | -0.08 (-0.52, 0.89) | | 0.03 (-0.41, 0.87) | | | | -0.10 (-0.33, 0.63) | | -0.38 (-0.96, 0. 20) | | **-0.21 (-0.42, -0.05)**^3^ | | | -0.18 (-0.47, 0.10) | |  |

*Z-scores for birth length, weight, and head circumference were calculated using WHO child growth standards that account for newborns sex and chronological age (non-gestational age-adjusted data); Arm 1: Received nutrition supplement before and during pregnancy (preconception to delivery); Arm 2: Received nutrition supplement only during pregnancy (from 12 weeks of gestation to delivery); Arm 3: No nutrition supplement (control Arm); Hb: Hemoglobin; 95% CIs: 95% Confidence intervals; WHO: World Health Organization.*

*^1^Generalized estimated equations were used to calculate mean differences in the Z-scores birth length, weight, and head circumference and their 95% CIs after accounting for cluster correlations and adjusting for maternal age, parity, education, socioeconomic status, and pre-pregnancy body mass index; Using Bonferroni method, adjustment for multiple testing was done for three arms, three outcomes and six levels of effect modifier and multiple comparisons were evaluated at a significance level of 0.0009.*

^2^*p-value <0.0009.*

^3^*p-value: <0.05 but >0.0009; Bolded values indicate significant differences*

^4^*There were not enough women (n=6) in Hb category of ≥130g/L to calculate adjusted mean differences across three Arms for India (See Supplemental Table 1).*

^5^*There were not enough women (n=2) in Hb category of 80-89g/L to calculate the mean differences for the Democratic Republic of Congo (DRC) (See Supplemental Table 2).*

| **Supplemental Table 5: Pooled mean difference (95% CIs) in Z-scores for birth length, weight, and head circumference-for-gestational age between the randomized arms: Stratified by pre-pregnancy hemoglobin (g/L) categories- (Gestational age adjusted data: INTERGROWTH-21st fetal growth standards; n =1,456)** | | | | | | |
| --- | --- | --- | --- | --- | --- | --- |
|  | ***Arm 1*** | ***Arm 2*** | ***Arm 3*** | ***Arm 1 vs. Arm 3*** | ***Arm 1 vs. Arm 2*** | ***Arm 2 vs. Arm 3*** |
|  | ***Pooled mean ±SD*** | | | ***Pooled mean differences in Z-scores*^1^**  ***(95% CIs)*** | | |
| **Birth length-for-gestational age Z-scores** | | | | | | |
| Hb: 80-89g/L | -0·89 ±1·02 | -0·56 ±0·94 | -1·15 ±1·05 | 0·27 (-4·23, 4·86) | -0·33 (-1·72, 0·89) | 0·60 (-1·83, 3·02) |
| Hb: 90-99 g/L | -0·58±0·96 | -0·90 ±1·05 | -0·99 ±1·14 | **0·42 (0·21, 0·61)** | 0·33 (-0·29, 0·90) | 0·09 (-0·34, 0·52) |
| Hb: 100-109 g/L | -0·52 ±0·91 | -0·67 ±1·11 | -0·85±1·03 | **0·34 (0·10, 0·60)** | 0·16 (-0·92, 1·36) | 0·18 (-0·66, 1·03) |
| Hb: 110-119 g/L | -0·61±0·92 | -0·63 ±1·04 | -0·69±1·21 | 0·08 (-0·17, 0·32) | 0·02 (-0·33, 0·49) | 0·06 (-0·52, 0·95) |
| Hb: 120-129 g/L | -0·93 ±1·07 | -0·96 ±1·10 | -0·95±0·97 | -0·12 (-0·44, 0·18) | -0·05 (-0·37, 0·22) | -0·07 (-0·33, 0·22) |
| Hb ≥130 g/L | -0·74 ±0·98 | -0·77±0·86 | -0·72±0·81 | -0·03 (-0·73, 0·68) | 0·02 (-1·70, 1·56) | -0·05 (-4·52, 4·41) |
| **Birth weight-for-gestational age Z-scores** | | | | | | |
| Hb: 80-89g/L | -1·13 ±0·91 | -0·90 ±1·02 | -1·32 ±0·93 | 0·18 (-4·31, 4·67) | -0·24 (-2·50, 2·01) | 0·42 (-1·02, 1·53) |
| Hb: 90-99 g/L | -0·89±0·97 | -1·20 ±0·89 | -1·26 ±0·93 | 0·38 (-1·63, 2·39) | **0·33 (0·16, 0·49)** | 0·05 (-1·87, 2·05) |
| Hb: 100-109 g/L | -1·03 ±0·98 | -1·12 ±1·02 | -1·38 ±0·85 | **0·36 (0·12, 0·58)** | 0·09 (-0·08, 0·17) | 0·27 (-0·02, 0·58) |
| Hb: 110-119 g/L | -0·97±0·98 | -0·93 ±0·81 | -0·98 ±0·85 | 0·005 (-0·21, 0·22) | -0·04 (-0·20, 0·46) | 0·05 (-0·67, 0·84) |
| Hb: 120-129 g/L | -0·87±0·87 | -0·75 ±0·85 | -0·72 ±0·96 | -0·15 (-0·50, 0·19) | -0·14 (-0·31, 0·03) | -0·01 (-0·38, 0·37) |
| Hb ≥130 g/L | -0·69 ±0·78 | -0·68 ±0·85 | -0·72 ±0·71 | 0·03 (-0·21, 0·27) | -0·01 (-1·65, 1·44) | 0·04 (-1·36, 1· 60) |
| **Birth head circumference-for-gestational age Z-scores** | | | | | | |
| Hb: 80-89g/L | -0·81 ±1·05 | -0·44±1·04 | -0·92 ±0·98 | 0·10 (-1·28, 1·48) | -0·37 (-0·61, 0·24) | 0·47 (-1·36, 2·31) |
| Hb: 90-99 g/L | -0·42 ±1·15 | -0·75±0·79 | -0·67±0·97 | 0·25 (-0·04, 0·51) | 0·34 (-0·17, 0·43) | -0·08 (-2·76, 2·60) |
| Hb: 100-109 g/L | -0·55 ±0·64 | -0·62±1·07 | -0·79± 1·16 | 0·23 (-0·71, 1·43) | 0·07 (-0·14, 0·42) | 0·17 (-0·35, 0·94) |
| Hb: 110-119 g/L | -0·73 ±1·00 | -0·70±0·97 | -0·69 ±1·19 | -0·04 (-0·70, 0·80) | -0·03 (-0·48, 0·60) | -0·004 (-0·58, 0·52) |
| Hb: 120-129 g/L | -0·44 ±1·24 | -0·36 ±0·99 | -0·19±1·04 | -0·25 (-1·26, -0·73) | -0·05 (-0·25, 0·30) | -0·21 (-1·24, 0·83) |
| Hb ≥130 g/L | -0·48 ±0·95 | -0·21 ±1·05 | -0·22 ±0·96 | -0·27 (-3·72, 3·02) | -0·27 (-1·64, 1·13) | 0·01 (-1·34 ,1·42) |

*Hb: Hemoglobin; SD: standard deviation; 95% CIs: 95% Confidence intervals.*

*Arm 1: Received nutrition supplement before and during pregnancy (preconception to delivery); Arm 2: Nutrition supplement only during pregnancy (from 12 weeks of gestation to delivery); Arm 3: No nutrition supplement (control Arm).*

^1^*Using meta-analytic approach, pooled mean differences (95% CIs) in Z-scores for birth length, weight, and head circumference-for-gestational age were calculated by pooling site-specific results of effect measure modification from Pakistan, India, and Guatemala (Supplemental Tables:6-8) using INTERGROWTH-21st fetal growth standards (gestational age adjusted data).*

| **Supplemental Table 6:** **Adjusted mean differences in Z-scores for birth length, weight, and head circumference-for-gestational age between the randomized arms: Stratified by pre-pregnancy hemoglobin (g/L)- *(Pakistan; n = 456)*** | | | | | | |
| --- | --- | --- | --- | --- | --- | --- |
|  | **Arm 1** | **Arm 2** | **Arm 3** | **Arm 1 vs. Arm 3** | **Arm 1 vs. Arm 2** | **Arm 2 vs. Arm 3** |
|  | **Adjusted mean ±SD** | | | **Adjusted mean differences in Z-scores^1^**  **(95% CIs)** | | |
| **Birth length-for-gestational age Z-scores** | | | | | | |
| Hb: 80-89g/L | -0·70 ±1·22 | -0·49 ±1·23 | -1·07 ±1·11 | 0·38 (-0·04, 0·81) | -0·20 (-1·00, 0·73) | **0·58 (0·31, 1·45)**^3^ |
| Hb: 90-99 g/L | -0·76 ±1·04 | -1·13 ±1·31 | -1·47 ±1·01 | **0·71 (0·03, 1·47)**^3^ | 0·37 (-0·42, 0·77) | 0·34 (-0·53, 0·94) |
| Hb: 100-109 g/L | -0·81 ±0·84 | -0·71 ±1·04 | -1·28 ±1·16 | **0·48 (0·23, 0·73)**^2^ | -0·09 (-0·53, 0·35) | **0·57 (0·20, 0·94)**^3^ |
| Hb: 110-119 g/L | -0·67 ±1·05 | -0·72 ±1·07 | -0·76 ±1·36 | 0·09 (-0·53, 0·71) | 0·15 (-0·36, 0·40) | 0·07 (-0·53, 0·67) |
| Hb: 120-129 g/L | -0·99 ±1·32 | -1·21 ±1·21 | -1·18 ±1·13 | 0·19 (-0·29, 0·68) | 0·21 (-0·31, 0·73) | -0·02 (-0·42, 0·37) |
| Hb ≥130 g/L | -0·76 ±1·09 | -0·40 ±0·94 | -0·31 ±0·89 | -0·45 (-0·97, 0·84) | -0·36 (-0·71, 1·26) | -0·09 (-1·30, 0·62) |
| **Birth weight-for-gestational age Z-scores** | | | | | | |
| Hb: 80-89g/L | -0·96 ±0·94 | -0·64 ±1·40 | -1·16 ±0·76 | 0·20 (-0·21, 0·60) | -0·33 (-0·82, 0·90) | 0·53 (-0·05, 1·12) |
| Hb: 90-99 g/L | -0·76 ±0·91 | -1·18 ±1·12 | -1·31 ±0·89 | **0·57 (0·03, 1·10)**^3^ | 0·42 (-0·12, 0·81) | 0·15 (-0·64, 0·33) |
| Hb: 100-109 g/L | -0·88 ±1·05 | -1·01 ±1·09 | -1·27 ±0·81 | **0·39 (0·02, 0·73)**^3^ | 0·13 (-0·23, 0·46) | 0·26 (-0·18, 0·71) |
| Hb: 110-119 g/L | -0·96 ±0·95 | -0·94 ±0·82 | -0·92 ±0·77 | -0·01 (-0·58, 0·56) | 0·04 (-0·47, 0·56) | -0·06 (-0·46, 0·35) |
| Hb: 120-129 g/L | -0·75 ±1·01 | -0·66 ±0·92 | -0·70 ±1·12 | -0·06 (-0·47, 0·35) | -0·08 (-0·46, 0·28) | 0·03 (-0·38, 0·44) |
| Hb ≥130 g/L | -0·80 ±0·55 | -0·95 ±0·99 | -0·87 ±0·96 | 0·07 (-0·27, 0·41) | 0·13 (-0·34, 0·61) | -0·07 (-0·43, 0·30) |
| **Birth head circumference-for-gestational age Z-scores** | | | | | | |
| Hb: 80-89g/L | -0·87 ±1·09 | -0·67 ±1·05 | -1·04 ±1·04 | 0·17 (-0·23, 0·58) | -0·19 (-1·31, 0·40) | 0·36 (-0·06, 1·32) |
| Hb: 90-99 g/L | -0·63 ±0·74 | -0·95±1·24 | -1·03±1·22 | **0·41 (0·01, 0·84)**^3^ | 0·33 (-0·18, 0·84) | 0·08 (-0·95, 0·20) |
| Hb: 100-109 g/L | -0·48 ±1·15 | -0·68 ±0·92 | -0·85 ±1·02 | 0·38 (-0·02, 0·87) | 0·23 (-0·48, 0·78) | 0·15 (-0·52, 0·83) |
| Hb: 110-119 g/L | -0·96 ±1·10 | -0·95±1·12 | -0·92 ±1·14 | -0·05 (-0·67, 0·56) | -0·01 (-0·50, 0·49) | 0·01 (-0·65, 0·66) |
| Hb: 120-129 g/L | -0·40 ±1·16 | -0·39 ±1·08 | -0·21 ±1·52 | -0·20 (-0·55, 0·14) | -0·01 (-0·41, 0·38) | -0·19 (-0·55, 0·17) |
| Hb ≥130 g/L | -0·67 ±1·09 | -0·21 ±1·44 | -0·06 ±1·67 | **-0·67 (-1·17, -0·19)**^3^ | **-0·48 (-1·03, -0·06)**^3^ | -0·20 (-0·68, 0·30) |

*Hb: Hemoglobin; 95% CIs: 95% Confidence intervals; SD: standard deviation.*

*Z-scores for birth length, weight, and head circumference -for-gestational age were calculated using INTERGROWTH-21st fetal growth standards (gestational age adjusted data).*

*Arm 1: Received nutrition supplement before and during pregnancy (preconception to delivery); Arm 2: Nutrition supplement only during pregnancy (from ~ 12 weeks of gestation to delivery); Arm 3: No nutrition supplement (control Arm).*

^1^*Generalized estimated equations were used to calculate mean differences and their 95% CIs after accounting for cluster correlations and adjusting for maternal age, parity, education, socioeconomic status, and pre-pregnancy body mass index.*

*Using Bonferroni method, adjustment for multiple testing was done for three arms, three outcomes and six levels of effect modifier and multiple comparisons were evaluated at a significance level of 0·0009.*

^2^*p-value <0·0001.*

^3^*p-value: <0·05 but >0·0009; bolded values indicate significant differences.*

**Supplemental Table 7: Adjusted mean differences in Z-scores for birth length, weight, and head circumference-for-gestational age between randomized arms: Stratified by pre-pregnancy hemoglobin (g/L)- (India, n = 507)**

|  | **Arm 1** | **Arm 2** | **Arm 3** | **Arm 1 vs. Arm 3** | **Arm 1 vs. Arm 2** | **Arm 2 vs. Arm 3** | |
| --- | --- | --- | --- | --- | --- | --- | --- |
|  | **Adjusted mean ±SD** | | | **Adjusted mean differences in Z-scores^1^**  **(95% CIs)** | | | |
| **Birth length-for-gestational age Z-scores** | | | | | | | |
| Hb: 80-89g/L | -0·94 ±0·80 | -0·49±0·73 | -0·96±1·11 | 0·02 (-0·34, 0·38) | **-0·45 (-0·77, -0·14)**^2^ | **0·47 (0·17, 0·77)**^2^ | |
| Hb: 90-99 g/L | -0·47 ±0·91 | -0·78±0·88 | -0·86 ±1·14 | **0·40 (0·14, 0·66)**^2^ | **0·32 (0·09, 0·55)**^2^ | 0·08 (-0·15, 0·31) | |
| Hb: 100-109 g/L | -0·51 ±0·67 | -0·66 ±1·08 | -0·67 ±1·18 | 0·16 (-0·25, 0·58) | 0·15 (-0·06, 0·37) | 0·01 (-0·50, 0·52) | |
| Hb: 110-119 g/L | -0·71 ±0·99 | -0·64 ±1·18 | -0·83 ±0·99 | 0·11 (-0·25, 0·47) | -0·06 (-0·45, 0·33) | 0·17 (-0·04, 0·40) | |
| Hb: 120-129 g/L | -0·74 ±1·09 | -0·98 ±1·22 | -0·83±1·21 | 0·09 (0·29, 0·68) | 0·25 (-0·01, 0·51) | -0·16 (-0·78, 0·46) | |
| Hb ≥130 g/L^3^ | Not defined | Not defined | Not defined | Not defined | Not defined | Not defined | |
| **Birth weight-for-gestational age Z-scores** | | | | | | | |
| Hb: 80-89g/L | -1·36 ±0·89 | -0·99 **±**0·73 | -1·22 ±1·08 | -0·14 (-0·47, 0·20) | -0·36 (-0·91, 0·20) | **0·22 (0·01, 0·44)**^2^ |  |
| Hb: 90-99 g/L | -0·92 ±1·01 | -1·25 ±0·74 | -1·42 ±0·89 | **0·52 (0·18, 0·84)**^2^ | **0·33 (0·04, 0·60)**^2^ | 0·19 (-0·03, 0·42) |  |
| Hb: 100-109 g/L | -1·12 ±0·93 | -1·19 ±1·02 | -1·42 ±0·88 | 0·30 (-0·09, 0·67) | 0·08 (-0·39, 0·54) | 0·22 (-0·07, 0·49) |  |
| Hb: 110-119 g/L | -1·00 ±1·02 | -1·23 ±0·87 | -1·11 ±1·04 | 0·10 (-0·24, 0·45) | 0·23 (-0·11, 0·60) | -0·13 (-0·48, 0·22) |  |
| Hb: 120-129 g/L | -1·40 ±0·96 | -1·46 ±0·99 | -0·86 ±1·08 | -0·51 (-0·50, 0·17) | -0·01 (-0·68, 0·64) | -0·49 (-1·45, 0·45) |  |
| Hb ≥130 g/L^3^ | Not defined | Not defined | Not defined | Not defined | Not defined | Not defined |  |
| **Birth head circumference-for-gestational age Z-scores** | | | | | | | |
| Hb: 80-89g/L | -0·87±1·02 | -0·43**±**1·04 | -0·79±0·92 | -0·07 (-0·71, 0·56) | -0·42 (-0·80, -0·04)^2^ | 0·34 (-0·29, 0·98) |  |
| Hb: 90-99 g/L | -0·38 ±1·23 | -0·68 ±0·71 | -0·76 ±0·93 | **0·37 (0·12, 0·62)**^2^ | **0·30 (0·10, 0·50)**^2^ | 0·07 (-0·18, 0·33) |  |
| Hb: 100-109 g/L | -0·47 ±0·88 | -0·77 ±0·87 | -0·78 ±1·18 | 0·32 (-0·17, 0·82) | 0·32 (-0·17, 0·82) | -0·01 (-0·37, 0·35) |  |
| Hb: 110-119 g/L | -0·62 ±1·09 | -0·69 ±0·98 | -0·63 ±1·15 | 0·01 (-0·56, 0·57) | 0·05 (-0·50, 0·60) | -0·04 (-0·43, 0·35) |  |
| Hb: 120-129 g/L | -0·67 ±1·12 | -0·79 ±0·97 | -0·18 ±1·08 | -0·77 (-1·60, 0·06) | 0·04 (-0·10, 0·18) | **-0·81 (-1·54, 0·08)**^2^ |  |
| Hb ≥130 g/L^3^ | Not defined | Not defined | Not defined | Not defined | Not defined | Not defined |  |

*Hb: Hemoglobin; 95% CIs: 95% Confidence intervals; SD: standard deviation.*

*Z-scores for birth length, weight, and head circumference-for-gestational age were calculated using INTERGROWTH-21st fetal growth standards (gestational age adjusted data).*

*Arm 1: Received nutrition supplement before and during pregnancy (preconception to delivery); Arm 2: Nutrition supplement only during pregnancy (from ~ 12 weeks of gestation to delivery); Arm 3: No nutrition supplement (control Arm).*

^1^*Generalized estimated equations were used to calculate mean differences and their 95% CIs after accounting for cluster correlations and adjusting for maternal age, parity, education, socioeconomic status, and pre-pregnancy body mass index.*

*Using Bonferroni method, adjustment for multiple testing was done for three arms, three outcomes and five levels of effect modifier and multiple comparisons were evaluated at a significance level of 0·001.*

^2^*p-value <0·05 but >0·001; bolded values indicate significant differences.*

*^3^There were not enough women (n=5) in this category to calculate adjusted mean differences across three Arms.*

| **Supplemental Table 8: Adjusted mean differences in Z-scores for length, weight, and head circumference-for-gestational age between randomized arms: Stratified by pre-pregnancy hemoglobin (g/L)*- (Guatemala, n = 493)*** | | | | | | |
| --- | --- | --- | --- | --- | --- | --- |
|  | **Arm 1** | **Arm 2** | **Arm 3** | **Arm 1 vs. Arm 3** | **Arm 1 vs. Arm 2** | **Arm 2 vs. Arm 3** |
|  | **Adjusted Mean ±SD** | | | **Adjusted mean differences in Z-scores (95% CIs)**^1^ | | |
| **Birth length-for-gestational age Z-scores** | | | | | | |
| Hb: 80-89g/L^4^ | Not defined | Not defined | Not defined | Not defined | Not defined | Not defined |
| Hb: 90-99 g/L^4^ | 1·09 ±NA | -1·20 ±0·54 | -1·71±0·78 | **2·81 (2·47, 3·15)**^2^ | **2·30 (1·99, 2·58)**^2^ | **0·52 (0·20, 0·84)**^2^ |
| Hb: 100-109 g/L | -0·88±0·30 | -1·56±0·70 | -1·44 ±0·25 | **0·56 (0·30, 0·82)**^2^ | **0·70 (0·45, 0·64)**^2^ | -0·13 (-0·38, 0·11) |
| Hb: 110-119 g/L | -0·69 ±1·02 | -0·40 ±0·77 | -0·92±0·76 | 0·22 (-0·47, 0·92) | -0·32 (-0·92, 0·28) | **0·54 (0·02, 1·06)** ***^ǂ^*** |
| Hb: 120-129 g/L | -0·95 ±0·79 | -0·73 ±0·90 | -0·78 ±0·75 | -0·16 (-0·56, 0·27) | -0·21 (-0·64, 0·21) | 0·05 (-0·29, 0·39) |
| Hb ≥130 g/L | -0·84 ±0·96 | -0·63 ±0·85 | -0·80 ±0·80 | -0·04 (-0·16, 0·12) | **-0·21 (-0·38, -0·03)**^2^ | **0·17 (0·05, 0·32) *^ǂ^*** |
| **Birth weight -for-gestational age Z-scores** | | | | | | |
| Hb: 80-89g/L^4^ | Not defined | Not defined | Not defined | Not defined | Not defined | Not defined |
| Hb: 90-99 g/L^4^ | 1·55 ±NA | -0·63 ±0·38 | -1·70 ±0·92 | **2·23 (2·17, 4·28)**^2^ | **1·18 (0·43, 1·47)**^2^ | **1·05 (0·05, 2·05)**^2^ |
| Hb: 100-109 g/L | -0·75 ±0·92 | -0·76 ±0·37 | -1·25 ±0·32 | 0·50 (-0·08, 1·07) | 0·01 (-0·20, 0·20) | 0·49 (-0·02, 1·01) |
| Hb: 110-119 g/L | -0·49 ±0·96 | -0·50 ±0·58 | -0·92 ±0·73 | 0·43 (-0·08, 1·07) | 0·01 (-0·47, 0·48) | **0·42 (0·11, 0·75)^3^** |
| Hb: 120-129 g/L | -0·84 ±0·68 | -0·64 ±0·67 | -0·67 ±0·78 | -0·16 (-0·50, 0·17) | -0·18 (-0·43, 0·05) | 0·02 (-0·28, 0·33) |
| Hb ≥130 g/L | -0·77 ±0·80 | -0·61 ±0·84 | -0·80 ±0·67 | 0·02 (-0·13, 0·19) | **-0·17 (-0·32, -0·01)**^2^ | 0·19 (0·05, 0·33) |
| **Birth head circumference-for-gestational age Z-scores** | | | | | | |
| Hb: 80-89g/L^4^ | Not defined | Not defined | Not defined | Not defined | Not defined | Not defined |
| Hb: 90-99 g/L^4^ | 1·47 ±NA | -0·68 ±1·09 | -0·41±0·50 | **1·91 (1·24, 2·58)**^3^ | **2·15 (1·48, 2·82)**^2^ | -0·24 (-0·87, 0·39) |
| Hb: 100-109 g/L | -0·30 ±0·78 | -0·18 ±0·99 | -1·03 ±0·41 | 0·70 (-0·55, 1·96) | -0·13 (-0·86, 1·46) | **0·84 (0·22, 1·47)**^3^ |
| Hb: 110-119 g/L | -0·25 ±1·01 | -0·11 ±0·70 | -0·44 ±1·32 | 0·22 (-0·68, 1·12) | -0·14 (-0·67, 0·40) | 0·36 (-0·26, 0·99) |
| Hb: 120-129 g/L | -0·42 ±1·03 | -0·16 ±0·89 | -0·13 ±0·92 | -0·30 (-0·80, 0·20) | -0·27 (-0·80, 0·20) | -0·03 (-0·41, 0·34) |
| Hb ≥130 g/L | -0·37 ±0·92 | -0·17±1·00 | -0·24 ±0·83 | -0·13 (-0·31, 0·04) | -0·20 (-0·41, 0·02) | 0·09 (-0·06, 0·26) |

*Hb: Hemoglobin; 95% CIs: 95% Confidence intervals; SD: standard deviation.*

*Z-scores for birth length, weight, and head circumference-for-gestational age were calculated using INTERGROWTH-21st fetal growth standards (gestational age adjusted data).*

*Arm 1: Received nutrition supplement before and during pregnancy (preconception to delivery); Arm 2: Nutrition supplement only during pregnancy (from ~ 12 weeks of gestation to delivery); Arm 3: No nutrition supplement (control Arm).*

^1^*Generalized estimated equations were used to calculate mean differences and their 95% CIs after accounting for cluster correlations and adjusting for maternal age, parity, education, socioeconomic status, and pre-pregnancy body mass index.*

*Using Bonferroni method, adjustment for multiple testing was done for three arms, three outcomes and six levels of effect modifier and multiple comparisons were evaluated at a significance level of 0·0009.*

^2^ *P-value< 0·0001.*

^3^ *p-value: <0·05 but >0·001; bolded values indicate significant differences.*

^4^*There were very few women (n=5 and n=7) in the two categories and were not included in the pooled analysis due to unstable mean differences. NA: Not applicable because SD could not be calculated due to one woman in Arm 1 for Hb 90-99g/L.*
