# Supplementary material for: Women with moderate anaemia prior to conception benefited most from nutrition interventions: a secondary analysis of the Women First preconception maternal nutrition trial
Source: BMJ Glob Health. 2026 Jan 23;11(1):e020160. doi: 10.1136/bmjgh-2025-020160 (PMC12853505; doi:10.1136/bmjgh-2025-020160)
Supplement: online supplemental file 2 [file bmjgh-11-1-s002.docx]

### BMJ Global Health Author Reflexivity Statement

Adapted from Morton, B., Vercueil, A., Masekela, R., Heinz, E., Reimer, L., Saleh, S., Kalinga, C., Seekles, M., Biccard, B., Chakaya, J., Abimbola, S., Obasi, A. and Oriyo, N. (2022), Consensus statement on measures to promote equitable authorship in the publication of research from international partnerships. Anaesthesia, 77: 264-276. <https://doi.org/10.1111/anae.15597>

| **Study conceptualisation** | |
| --- | --- |
| 1. How does this study address local research and policy priorities? | By evaluating how preconception nutrition interventions benefit women with varying anemia severity, the research provides evidence relevant to national strategies on maternal nutrition, anemia reduction, and newborn health. The findings support more targeted allocation of nutrition resources and align with policy efforts in LMICs to identify and prioritize high-risk women before pregnancy. |
| 1. How were local researchers involved in study design? | This study was developed through a collaborative partnership involving researchers from both high-income and low- and middle-income countries. Local investigators in Pakistan, India, Guatemala, and the Democratic Republic of Congo played central roles in shaping the study design. The lead author, Sumera Aziz Ali, based in Pakistan, led the study’s conceptualization, analytic approach, and manuscript development. Site investigators contributed extensively to protocol development, field implementation, and interpretation of results. |
| **Research management** | |
| 1. How has funding been used to support the local research team(s)? | Overall, the funding ensured that local teams were equipped to lead day-to-day operations and contribute meaningfully to the research. Funding was used to directly strengthen the capacity of the local research teams across participating sites. This included support for personnel salaries, training workshops, and skill-building activities. Resources were also allocated for transportation and field logistics to enable effective study implementation and monitoring at the community level. |
| **Data acquisition and analysis** | |
| 1. How are research staff who conducted data collection acknowledged? | Research staff who carried out data collection are acknowledged through several mechanisms. They are formally recognized in the Acknowledgements section of the manuscript. Additionally, all field staff also received appropriate compensation and were provided with training, supervision, and opportunities to build research skills throughout the project. |
| 1. How have members of the research partnership been provided with access to study data? | Members of the research partnership have been granted access to study data through a structured and transparent data-sharing process. All participating sites were provided access to their own site-level data during the study for monitoring, quality assurance, and analysis discussions. The fully de-identified dataset is available to all partners through the National Institute of Child Health and Human Development (NICHD) Data and Specimen Hub (DASH). This ensures that every collaborating institution, regardless of country, can access the data for secondary analyses, capacity building, and future research initiatives. |
| 1. How were data used to develop analytical skills within the partnership? | Local and international team members were actively engaged in data management, cleaning, and analysis under the supervision of experienced investigators. Training workshops and mentorship sessions strengthened skills in statistical methods, data interpretation, and analytical software use. Local researchers applied the study data to conduct site-specific analyses, leading to multiple publications. |
| **Data interpretation** | |
| 1. How have research partners collaborated in interpreting study data? | Research partners from all study sites collaborated through regular meetings and workshops to review and interpret study findings. Local investigators contributed contextual knowledge, while international team members provided methodological guidance, ensuring that interpretations were accurate, culturally appropriate, and relevant to local research and policy priorities. |
| **Drafting and revising for intellectual content** | |
| 1. How were research partners supported to develop writing skills? | Research partners were supported to develop writing skills through opportunities to analyse study data and draft manuscripts as first or co-authors. Mentorship and feedback from experienced international investigators guided revisions, strengthened scientific writing, and enhanced publication readiness. |
| 1. How will research products be shared to address local needs? | Research findings, site-specific analyses, and publications will be shared with local stakeholders, including health authorities, community organizations, and participating health care facilities. Summaries and policy briefs will be provided in accessible formats to inform local programs and support evidence-based interventions for maternal and child health. |
| **Authorship** | |
| 1. How is the leadership, contribution and ownership of this work by LMIC researchers recognised within the authorship? | Leadership, contributions, and ownership by LMIC researchers are recognized through authorship that reflects their roles in study design, data collection, analysis, and manuscript preparation. Authorship decisions followed International Committee of Medical Journal Editors (ICMJE) criteria to ensure all contributors received appropriate credit for their work. |
| 1. How have early career researchers across the partnership been included within the authorship team? | Early career researchers across the partnership were actively included in the authorship team. For example, the first author was given the opportunity to clean, manage, and analyse the study data, draft the initial manuscript, receive feedback from mentors, and independently guide it through multiple stages to final submission. Local junior investigators also provided feedback on site-specific analyses and contributed to manuscript reviwe. |
| 1. How has gender balance been addressed within the authorship? | Gender balance was considered in authorship decisions, ensuring that women researchers at both local and international sites were included in key roles such as study design, data analysis, and manuscript writing. Efforts were made to provide equitable opportunities for leadership, first authorship, and co-authorship across genders. |
| **Training** | |
| 1. How has the project contributed to training of LMIC researchers? | The project contributed to training LMIC researchers through hands-on experience in data collection, management, and analysis, as well as manuscript preparation. Workshops, mentorship, and collaborative review of study findings strengthened research, analytical, and scientific writing skills, building long-term capacity at participating sites. |
| **Infrastructure** | |
| 1. How has the project contributed to improvements in local infrastructure? | The project contributed to improvements in local research infrastructure by providing equipment, resources, and logistical support necessary for data collection and monitoring. Investments in training, point-of-care devices, and local laboratory capacity strengthened the ability of participating sites to conduct high-quality research and support future studies. |
| **Governance** | |
| 1. What safeguarding procedures were used to protect local study participants and researchers? | Safeguarding procedures included obtaining written informed consent from all participants, approval from institutional and local ethics committees, and oversight by a data monitoring committee. Study staff received training on ethical conduct, participant confidentiality, and safety monitoring to protect both participants and researchers throughout the study. |
